# NAD^+^ Acts as a Protective Factor in Cellular Stress Response to DNA Alkylating Agents

**DOI:** 10.3390/cells12192396

**Published:** 2023-10-02

**Authors:** Joanna Ruszkiewicz, Ylea Papatheodorou, Nathalie Jäck, Jasmin Melzig, Franziska Eble, Annika Pirker, Marius Thomann, Andreas Haberer, Simone Rothmiller, Alexander Bürkle, Aswin Mangerich

**Affiliations:** 1 Molecular Toxicology Group, Department of Biology, University of Konstanz, 78457 Konstanz, Germany; 2 Bundeswehr Institute of Pharmacology and Toxicology, 80937 Munich, Germany; simone1rothmiller@bundeswehr.org; 3 Nutritional Toxicology, Institute Nutritional Science, University of Potsdam, 14469 Potsdam, Germany

**Keywords:** nicotinamide adenine dinucleotide, NAD booster, mustard agents, nicotinamide riboside, DNA damage, sulfur mustard, poly(ADP-ribosylation), PARP

## Abstract

Sulfur mustard (SM) and its derivatives are potent genotoxic agents, which have been shown to trigger the activation of poly (ADP-ribose) polymerases (PARPs) and the depletion of their substrate, nicotinamide adenine dinucleotide (NAD^+^). NAD^+^ is an essential molecule involved in numerous cellular pathways, including genome integrity and DNA repair, and thus, NAD^+^ supplementation might be beneficial for mitigating mustard-induced (geno)toxicity. In this study, the role of NAD^+^ depletion and elevation in the genotoxic stress response to SM derivatives, i.e., the monofunctional agent 2-chloroethyl-ethyl sulfide (CEES) and the crosslinking agent mechlorethamine (HN2), was investigated with the use of NAD^+^ booster nicotinamide riboside (NR) and NAD^+^ synthesis inhibitor FK866. The effects were analyzed in immortalized human keratinocytes (HaCaT) or monocyte-like cell line THP-1. In HaCaT cells, NR supplementation, increased NAD^+^ levels, and elevated PAR response, however, did not affect ATP levels or DNA damage repair, nor did it attenuate long- and short-term cytotoxicities. On the other hand, the depletion of cellular NAD^+^ via FK866 sensitized HaCaT cells to genotoxic stress, particularly CEES exposure, whereas NR supplementation, by increasing cellular NAD^+^ levels, rescued the sensitizing FK866 effect. Intriguingly, in THP-1 cells, the NR-induced elevation of cellular NAD^+^ levels did attenuate toxicity of the mustard compounds, especially upon CEES exposure. Together, our results reveal that NAD^+^ is an important molecule in the pathomechanism of SM derivatives, exhibiting compound-specificity. Moreover, the cell line-dependent protective effects of NR are indicative of system-specificity of the application of this NAD^+^ booster.

## 1. Introduction 

Sulfur mustard (SM, bis[2-chloroethyl] sulfide), also known as mustard gas, is a chemical warfare agent, initially used during WWI. Despite being banned by the Chemical Weapons Convention (CWC), it is still occasionally released in asymmetric conflicts or terrorist attacks. Furthermore, old SM depots pose a threat due to potential release and accidental exposure [1]. SM has strong vesicant and blister-forming effects on skin and eyes. When absorbed, it leads to pulmonary, cardiovascular, gastrointestinal, or immunological diseases, and it also increases the risk of cancer [2,3]. To date, no effective therapy against SM-induced pathology is available [3,4]. Furthermore, SM-derivatives, i.e., nitrogen mustards, such as chlorambucil or melphalan, display cytostatic properties that are applied in chemotherapy [5]. Therefore, studying the molecular mechanisms of mustard agent toxicity may not only help mitigate the symptoms of SM exposure but also contribute to more efficient and safer anti-cancer treatments.

Mechanistically, SM is a bi-functional alkylating agent that can alkylate different molecules impairing cellular functions. For instance, the alkylation of DNA (particularly guanine nucleotides) leads to the formation of mono- and bi-adducts, promoting the formation of intra- and inter-strand crosslinks (ICLs), contributing to SM toxicity [6,7]. Additionally, other types of DNA damage, including DNA strand breaks (DSBs), which are formed in the process of DNA repair, can also be found following SM exposure [8]. DNA damage can halt proliferation and lead to cell death; however, in the case of cell survival, the damaged DNA might lead to the formation of mutations during cell division and thus promote carcinogenesis [9]. This can be counteracted by several specialized cellular DNA repair pathways. One of the earliest events in the DNA damage response is the recruitment of poly(ADP-ribose) polymerase 1 (PARP1) to the site of damage [10]. PARP1 is a member of the ‘diphtheria toxin–like ADP ribosyl transferases’ (ARTDs, also called poly-ADP-ribose polymerases (PARPs)), a family of enzymes that catalyze the formation of polymer poly(ADP-ribose) (PAR) using the oxidized form of nicotinamide adenine dinucleotide (NAD^+^) as a substrate [10]. The formation of PAR facilitates the repair of damaged DNA and prevents carcinogenicity, but when activated on a large scale (due to massive DNA damage as in SM exposure), it leads to the depletion of cellular NAD^+^ levels [11]. NAD^+^ is an essential molecule in major biological processes; thus, its depletion may impair multiple cellular functions. NAD^+^ depletion was shown for several alkylating agents, such as methyl methanesulfonate (MMS) [12], N′-nitro-N-nitroso-N-methylguanidine (MNNG) [13], temozolomide [14], and sulfur mustard [15]. A mustard-induced decrease in the NAD^+^ pool disrupts glycolysis and produces an ATP deficit, which, in turn, might lead to cellular death via necrosis [15]. Additionally, NAD^+^ depletion might impair redox homeostasis, leading to oxidative-nitrosative stress (ONS) and mitochondrial dysfunction [16]; affect intracellular calcium signaling [17]; or activate inflammatory responses [18]. Thus, preventing the decline of cellular NAD^+^ levels appears to be a feasible strategy in addressing toxicity induced by mustard agent exposure [19].

**Figure 1:**
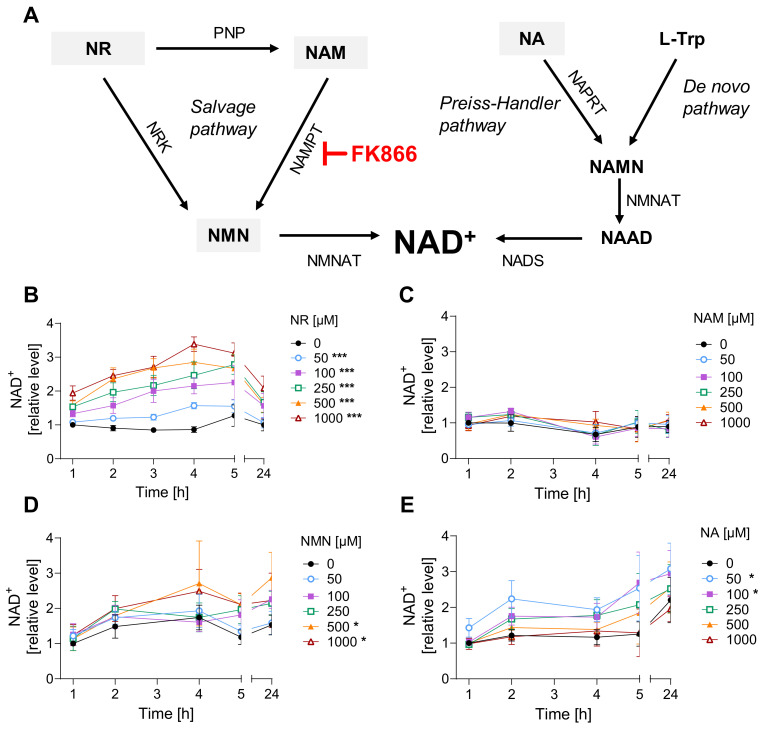
NR is the most efficient among tested NAD^+^ supplements. (**A**) NAD^+^ synthesis pathways. Abbreviations: L-Trp: L-tryptophane; NA: nicotinic acid; NAAD: nicotinate adenine dinucleotide; NAD^+^: nicotinamide adenine dinucleotide; NADS: NAD synthase; NAM: nicotinamide; NAMN: nicotinate mononucleotide; NAMPT: nicotinamide phosphoribosyltransferase; NAPRT: nicotinate phosphoribosyltransferase; NMN: nicotinamide mononucleotide; NMNAT: nicotinamide mononucleotide adenylyltransferase; NR: nicotinamide riboside; NRK: nicotinamide riboside kinases; PNP: purine nucleoside phosphorylase. (**B**–**E**) NAD^+^ levels upon treatment with precursors. HaCaT cells were harvested immediately after exposure for 1, 2, 3, 4, 5, and 24 h with NR ((**B**), n = 3–4), NAM ((**C**), n = 3–4), NMN ((**D**), n = 2–3), or NA ((**E**), n = 3). Cellular NAD^+^ levels were measured via enzymatic cycling assay, and data were normalized to the control (0 µM) at 1 h. The results were expressed as mean ± SEM and analyzed by two-way ANOVA with Tukey’s multiple comparison test. * *p* < 0.05, *** *p* < 0.001 vs. “0 µM”.

In mammals, NAD^+^ is predominantly synthesized via the salvage pathway, which converts nicotinamide (NAM) into nicotinamide mononucleotide (NMN) by NAM phosphoribosyl transferase (NAMPT), followed by the NMN adenylyl transferase (NMNAT)-dependent conversion of NMN into NAD^+^. As NAM is a by-product of NAD^+^ consumption by ARTDs, sirtuins (SIRTs), and NAD^+^ glycohydrolases, it provides a constant precursor to replenish the cofactor (reviewed in [20,21]). Additionally, nicotinamide riboside (NR), which can be supplemented through diet, might be converted into NMN by NR kinase (NRK) and integrated into the salvage pathway (Figure 1A). On the other hand, NMN can be converted to NR in the extracellular space via ecto-5′-nucleotidase (CD73) [22]. Additionally, NAD^+^ can be synthesized from nicotinic acid (NA) in the Preiss-Handler pathway or from L-tryptophan through the kynurenine pathway (de novo) (Figure 1A) [20]. Intracellular NAD^+^ levels can also be regulated by the activity of nicotinamide N-methyltransferase that methylates nicotinamide, preventing its use in the NAD^+^ salvage pathway [23]. To date, only NA and NAM have been investigated regarding mustard agent exposure, providing partial but not sufficient protection against cytotoxicity or histopathological damage, as we reviewed elsewhere [19]. Since NAD^+^ is not cell-permeable and therefore cannot be used as a supplement on its own, in the present study, we examined the effects of NR supplementation, which shows better bioavailability and tolerance in comparison to NA or NAM, as demonstrated in recent studies [24,25]. Moreover, to further examine the functional role of NAD^+^ in mustard-related toxicity, NAD^+^ depletion was induced via the salvage pathway inhibitor FK866 [26] prior to genotoxic treatments. Due to restrictions by the CWC, SM was replaced in our experiments with surrogate substances, such as a mono-functional alkylating agent, 2-chloroethyl ethyl sulfide (CEES), which displays similar vesicant and cytotoxic properties, but at 100-fold higher concentrations than SM. Another compound is the bi-functional nitrogen mustard bis(2-chloroethyl)methylamine (HN2), which has slightly lower toxicity than SM [27]. Our group previously demonstrated that both CEES and HN2 showed (distinct) similarities to SM-induced toxicity in human keratinocytes HaCaT [28]. In the present study, we continued this approach and applied both compounds side by side, while investigating the role of NAD^+^ and effects of NAD^+^ boosting in mustard-induced genotoxicity.

## 2. Materials and Methods 

### 2.1. Cell Culture

As the skin is one of the primary targets of SM exposure, we used the human keratinocyte cell line HaCaT as an experimental model system of human keratinocytes [29] (a kind gift by Petra Boukamp, formerly German Research Cancer Center, Heidelberg, Germany, DKFZ, generator and primary source of the cell line). Cells were cultured in DMEM medium (41966029, Gibco^TM^, Thermo Fisher Scientific, Waltham, MA, USA). Moreover, human monocytic leukemia cell line THP-1 [30] (accession number THP-1 TIB-202 at ATCC database) was tested, and these cells were grown in Roswell Park Memorial Institute (RPMI) 1640 medium (21875, Gibco^TM^, Thermo Fisher Scientific). Cells were tested for mycoplasma contamination, and experiments were performed on cells between passages 3 and 15 after thawing. Growth media were supplemented with 10% fetal calf serum (Bio&Sell, Feucht, Germany) and 1% penicillin-streptomycin (Gibco^TM^, Thermo Fisher Scientific). Cells were cultured at standard conditions: 37 °C, 95% humidity, and 5% CO_2_. For detachment, HaCaT cells were incubated with 0.25% trypsin-EDTA (Gibco^TM^, Thermo Fisher Scientific) for 5–10 min at 37 °C.

### 2.2. Treatment with Chemicals 

The NAD^+^ precursors: nicotinamide (NAM; Sigma-Aldrich, St. Louis, MO, USA), nicotinic acid (NA; Sigma-Aldrich), nicotinamide riboside (NR; MedChem Express, Monmouth Junction, NJ, USA), and nicotinamide mononucleotide (NMN; Cayman Chemical, Ann Arbor, MI, USA) were prepared in MiliQ-H_2_O stock solutions (10 mM) and stored at −80 °C. Compounds were added to the culture medium (final concentrations 0.05–1 mM) and incubated for varying durations (1–24 h) under standard conditions.

The pharmacological compound daporinad (FK866; MedChem Express) was used to inhibit the salvage pathway enzyme, NAMPT, and thus decrease cellular NAD^+^ levels as a result of NAD^+^ turnover. Stock solutions of 10 mM FK866 were prepared in DMSO and stored at −80 °C. The compound was added to the cells in fresh culture medium both 24 h before and immediately after mustard treatment. Control samples were incubated with 0.5% (*v*/*v*) DMSO.

Premixes (100×) of CEES (Sigma-Aldrich) or HN2 (Sigma-Aldrich) solutions were freshly prepared in a solvent containing 95% EtOH and 0.5% HCl (*v*/*v*) directly before cell treatment as described previously [31]. Then, solutions were prepared in pre-warmed PBS (or culture medium) and immediately added to the cells for incubation at standard conditions. Controls were incubated with 1% solvent. Due to the high toxicity and carcinogenicity, these chemicals were handled with extreme caution, in the appropriate laboratory settings by well-trained personnel. Exposure to H_2_O_2_ (100 µM) was used as a positive control of genotoxicity and PARP1 activation. After toxic treatment, cells were washed twice with PBS and supplemented with fresh culture medium or harvested immediately for analysis.

### 2.3. Cellular NAD^+^ Levels

Cellular NAD^+^ levels were determined using an enzymatic cycling assay [32]. HaCaT cells were seeded at 0.8 or 0.4 × 10^6^ (for 1–6 h, or 24 h of NR exposure, respectively) per well in 6-well plates 24 h prior to the experiment. THP-1 cells (2 × 10^6^ cells/mL) were incubated in 12-well plates during the treatments. After the treatments, cells were harvested (including detached cells in culture media), resuspended in 0.5 mL cold PBS, and placed on ice. Subsequently, 24 µL of perchloric acid (3.5 M) was added, samples were incubated for 15 min on ice, and centrifuged (10 min, 1500× *g*, 4 °C). The supernatant was mixed with 350 µL of 0.33 M K_2_HPO_4_ (pH 7.5), incubated for 15 min on ice, snap-frozen in liquid nitrogen, and stored at −80 °C until NAD^+^ analysis. The pellet was stored at −20 °C until analysis of the total protein content. For the NAD^+^ assay, after thawing and centrifugation (10 min, 1500× *g*, 4 °C), 40 µL of each sample (in technical triplicates) was used for the enzymatic reaction in a 96-well plate format. To each sample, 160 µL of buffer (0.25 M H_3_PO_4_, 0.5 M NaOH) and 100 µL of the reaction mixture (0.34 M bicine-NaOH pH 8.0, 2.9 mg/mL BSA, 14.3 mM EDTA, 1.4 mM MTT, 1.7 M EtOH, 5.7 mM phenazine ethosulfate, 0.14 mg/mL ADH) were added. The reaction mixture was also added to wells containing 200 µL of NAD^+^ standard (Sigma-Aldrich) dissolved in the buffer (0.25 M H_3_PO_4_, 0.5 M NaOH) in a concentration range of 0.05–0.5 µM. After a 30 min incubation at 30 °C, absorption was measured at 550 nm with 690 nm as a reference wavelength in a microplate reader (Infinite M200 Pro, Tecan, Männedorf, Switzerland). The results enabled the calculation of NAD^+^ concentrations in the samples, which were subsequently normalized to the total protein level. For the protein assay, sample pellets were dissolved in 500 μL 0.1 M NaOH, and the protein level was determined via a Pierce^TM^ BCA Protein Assay Kit (Thermo Fischer Scientific, Waltham, MA, USA) according to the manufacturer’s protocol.

### 2.4. Cellular ATP Levels

To measure cellular ATP levels, HaCaT cells were seeded 0.2 × 10^6^ cells/well in 12-well plates 24 h before treatment. Cell samples were collected immediately after the treatment (0 h) or after 24 h incubation with fresh culture media. Harvested cells were centrifuged (5 min, 600× *g*, RT), the pellet was resuspended in fresh culture medium, and ATP levels were measured with the Cellular ATP Kit HTS (BioThema, Handen, Sweden) according to manufacturer’s protocol in a 96-well plate format. The luminescence signal was measured with a microplate reader (Infinite M200 Pro, Tecan). The values were normalized to protein levels measured via a Pierce^TM^ BCA Protein Assay Kit (Thermo Fischer Scientific, Waltham, MA, USA) according to the manufacturer’s protocol.

### 2.5. Annexin V/propidium Iodide Staining

To analyze cell death, HaCaT cells were seeded at 0.15 × 10^6^ cells/well in 12-well plates 24 h before the treatment. After the exposure to genotoxicants, cells were supplemented with fresh culture medium and incubated for 24 h. Thereafter, cells and culture medium containing detached cells were collected and centrifuged (200× *g*, 5 min, 4 °C). After washing with cold PBS and centrifugation, the cell pellet was resuspended in an Annexin binding buffer (ABB) (10 mM HEPES/NaOH, pH 7.4, 140 mM NaCl, 2.5 mM CaCl_2_). A drop of Annexin V-APC solution (Invitrogen, Waltham, MA, USA) and 5 µL of propidium iodide (PI) solution (Sigma-Aldrich) were added to 100 µL of each sample and incubated in the dark for 15 min. Subsequently, 1 mL of ABB was added, samples were assayed by flow cytometry (FACSLyric^TM^, BD Biosciences, Franklin Lakes, NJ, USA), and results were analyzed by FlowJo^TM^ v.10.9.0 software (BD Biosciences).

### 2.6. AlamarBlue Viability Assay

To assess FK866 cytotoxicity, HaCaT cells were seeded into 96-well plates (5 × 10^3^ cells/well) 24 h before the treatment with FK866 (0.5–5 nM) for 24 or 48 h. Additionally, repeated exposure was applied (24 + 24 h) where, after 24 h, culture medium with FK866 was renewed, and cells were incubated for an additional 24 h. Thereafter, the culture medium was removed, and the fresh medium containing 10% AlamarBlue reagent (Invitrogen) was added to each well, according to the manufacturer’s protocol. Samples were incubated in standard conditions for 4 h, and the fluorescence signal was measured at 550 nm excitation and 590 nm emission wavelength with a microplate reader (Infinite M200 Pro, Tecan). Results were expressed as relative fluorescence normalized to the control (0.5% DMSO).

AlamarBlue assay was also used to assess THP-1 viability following mustard exposure. Briefly, 2 × 10^6^ cells/mL growth medium were incubated with 200 µM NR for 4 h at standard cell culture conditions. After the NR pre-treatment, growth medium was replaced with fresh growth medium containing CEES or HN2, and cells were incubated for 30 min. After mustard exposure, cells were centrifugated (5 min, 200× *g*, RT) and washed twice with 1 mL PBS. Next, cells were resuspended in 1 mL growth medium containing or not 200 µM NR (post-treatment), transferred into 96-well plates in triplicates (4 × 10^3^ cells/well), and incubated for 20 h at standard cell culture conditions. Thereafter, AlamarBlue reagent was added to each well, according to the manufacturer’s protocol. Samples were incubated in standard conditions for 2 h, and the fluorescence signal was measured at 550 nm excitation and 590 nm emission wavelength with a microplate reader (Infinite M200 Pro, Tecan). Results were expressed as relative fluorescence normalized to the control.

### 2.7. Clonogenic Survival Assay 

Clonogenic survival assays were performed as described previously [28]. HaCaT cells were seeded at 3 × 10^6^ cells on 100 mm culture dishes 24 h before treatment with FK866 and/or NR. Next, cells were harvested, resuspended at 10^6^ cells/mL culture medium, and treated with genotoxicants. After incubation, 100 µL of each sample was transferred into 9.9 mL of culture medium, resulting in a cell titer of 10 cells/µL. A total of 100 µL of such a sample (containing 10^3^ cells) was seeded into new 6-well plates (three wells per sample) with fresh culture medium and incubated for 7 days in the presence or absence of NR and/or FK866. Then, the medium was removed, and colonies were fixed by 30 min incubation with 10% formaldehyde (Sigma-Aldrich), followed by 30 min staining with 0.1% crystal violet (Sigma-Aldrich). The wells were washed with PBS and air-dried before being counted using OpenCFU software [33]. For each sample, the average of technical triplicates was normalized to the control.

### 2.8. DNA Damage

The induction of DNA strand breaks following the treatment with CEES and H_2_O_2_ was assessed by the automated fluorimetric alkaline DNA unwinding assay (FADU), as described previously [34,35]. HaCaT cells were seeded at 3 × 10^6^ cells on 100 mm culture dishes 24 h before the treatment. After, the toxic exposure samples were harvested immediately (0 h) or incubated for a further 24 h in fresh culture medium. Harvested cells were centrifuged (10 min, 300× *g*, 4 °C), and the pellet was resuspended in suspension buffer (10 mM sodium phosphate buffer, pH 7.4; 0.25 M myo-inositol; 1 mM MgCl_2_) at a final cell titer of 4 × 10^6^ cells/mL. Then, with the use of a liquid handling device (Genesis RSP100, Tecan), the FADU protocol was executed. Initially, 70 µL of each sample was transferred in technical triplicates into 96-well plates and lysed with 70 µL of lysis solution (9 M urea, 10 mM NaOH, 2.5 mM 1,2-cyclohexanedinitrilotetraacetic acid, 0.05% SDS) for 12 min at 0 °C. Next, 140 µL of alkaline solution (42.5% lysis solution, 0.2 M NaOH) was added, and alkaline DNA unwinding was performed at 30 °C for 60 min. Unwinding was stopped with 140 µL of neutralization solution (1 M glucose, 14 mM 2-mercaptoethanol). Directly after incubation, at 22 °C for 30 min, 156 µL of SYBR Green solution (Thermo Fischer Scientific) (1:8333 *v*/*v* in H_2_O) was added, samples were mixed by automatic pipetting, and the fluorescence signal was measured at 492 nm excitation and 520 nm emission wavelength in a microplate reader (Infinite M200 Pro, Tecan). For each sample, an internal control of the DNA levels was included, where unwinding was prevented by the addition of a neutralization solution prior to the alkaline solution. The relative amount of double-stranded DNA was calculated as (fluorescence of sample/fluorescence of sample without unwinding) × 100%. The HN2-induced interstrand crosslinks (ICLs) were measured via a reversed FADU (rFADU) protocol [34]. Then, prior to handling by LHD, samples were irradiated with an X-ray (24.66 Gray). The procedure induced DNA strand breaks, with the outcome of double-stranded DNA on the sites of ICL formation. As a result, the relative fluorescence signal was proportional to the amount of ICLs. The results were normalized to the untreated control at the respective time points.

### 2.9. PAR Formation

The immunochemical detection of PAR formation was performed as previously described [28,31]. HaCaT cells were seeded at 0.3 × 10^6^ cells per well in 12-well plates, on sterile 18 mm glass coverslips 24 h before the treatment. Directly after the toxic exposure, cells were fixed with ice-cold methanol for 7 min at −20 °C. Next, samples were washed 3 × 10 min with PBS and permeabilized with 0.4% Triton X-100 in PBS for 3 min. Then, samples were incubated in blocking solution (BS), containing 5% (*w*/*v*) milk powder and 0.5% Tween-20 in PBS, for 1 h at RT, and incubated with anti-PAR antibody (10H) (1:300 in BS) [36] overnight at 4 °C in a humidified chamber. Next, after 3 × 10 min washing with PBS, coverslips were incubated with Alexa546-labeled secondary antibody (Invitrogen) (1:300 in BS) for 1 h at RT in the dark in a humidified chamber. To visualize nuclei, samples were washed 3 × 10 min with PBS and incubated with Hoechst 33342 dye (0.2 µg/mL in PBS) (Thermo Fischer Scientific) for 5 min at RT. Next, coverslips were washed 3 × 10 min with PBS and mounted on glass slides using Aqua PolyMount (Polysciences, Warrington, PA, USA). Images were acquired using the Zeiss Axio Obrerver Z1 epifluorescence microscope and Axiovision software. At least 200 nuclei per sample were automatically analyzed using KNIME v.4.5.0 software [37]. 

### 2.10. Statistical Analysis

All experiments were performed in replicate numbers as indicated in figure legends. Data were analyzed with GraphPad Prism v.9.2.0 software, using statistical tests as indicated.

## 3. Results

### 3.1. NR Does Not Affect Cellular Response to Genotoxic Stress in HaCaT

To identify the most efficient strategy of NAD^+^ boosting in HaCaT cells, we applied a selection of NAD^+^ boosters, i.e., NA, NAM, NR, and NMN, for up to 24 h to the cell culture medium. Among them, NR resulted in the highest increase in NAD^+^ levels, up to 3-fold, within 4 h of exposure (Figure 1B). Other NAD^+^ precursors, such as NMN (Figure 1D) or NA (Figure 1E), showed lower or, in the case of NAM (Figure 1C), almost no effect on cellular NAD^+^ levels. As concentrations >50µM of NR showed statistically significant effects on increasing NAD^+^ levels, 100 µM NR was applied in further experiments. Exposure to 100 µM NR for 3 h resulted in an approximately 2-fold increase in cellular NAD^+^ levels, which remained significantly higher than the untreated control for up to 3 h after replacing the supplemented growth media with the regular DMEM (Figure S1). Such an effect is of physiological relevance, as it was shown that a single 1000 mg NR dose applied orally to a healthy 52-year-old male resulted in a 2.3-fold increase of NAD^+^ levels in peripheral blood mononuclear cells (PBMCs) at 4 h post-ingestion [25]. In a larger study, repeated oral NR administration elevated NAD^+^ levels in human PBMCs on average by around 60% when compared to the placebo group [38].

**Figure 2:**
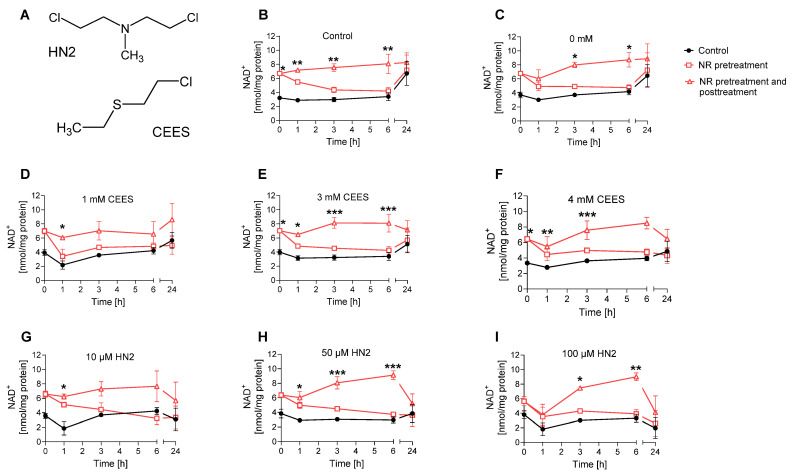
NR elevates NAD^+^ levels during genotoxic stress. (**A**) Chemical structures of 2-chloroethyl-ethyl sulfide (CEES) and bis(2-chloroethyl)methylamine (HN2). (**B**–**I**) HaCaT cells were supplemented with 100 μM NR for 3 h (pretreatment) and further treated with CEES or HN2 in PBS (“Control”) for 30 min; “0 mM” refers to solvent control. After exposure, cells were incubated with fresh growth medium ±100 μM NR (posttreatment) for up to 24 h or were harvested immediately (0 h). Cellular NAD^+^ levels were measured via enzymatic cycling assay and normalised to the total protein level measured by BCA. The results were expressed as mean ± SEM and analyzed by two-way ANOVA with Tukey’s multiple comparisons test (n = 3). * *p* < 0.05, ** *p* < 0.01, *** *p* < 0.001 vs. “Control”.

Subsequently, the effect of supplementation with 100 µM NR on cellular NAD^+^ levels was evaluated for up to 24 h following exposure to SM analogues, CEES and HN2. In line with previous observations in HaCaT keratinocytes [28], SM analogues did not lead to an immediate decrease of the cellular NAD^+^ levels; instead, a slight decrease was observed at 1 h after treatment. For example, 1 mM CEES induced a 27% decrease in cellular NAD^+^ levels 1 h post-exposure (Figure 2D); however, the effect was not dose-dependent, as higher CEES concentrations resulted in less NAD^+^ depletion (Figure 2E,F). For HN2, both 10 µM and 100 µM induced a 38% decrease within the first hour post-exposure (Figure 2H,I). The drop usually met control levels at later time points; however, at 24 h post-treatment for higher concentrations, such as 4 mM CEES (Figure 2F) or 100 µM HN2 (Figure 2I), the NAD^+^ levels were lower than those for control samples, by 25% and 70%, respectively. Usually, a slight decrease in NAD^+^ levels was also observed for NR-treated samples; however, the overall levels remained substantially higher. NR pre-treatment induced an increase in NAD^+^ levels (Figure 2), confirming the initial data (Figure 1B). Moreover, NR supplementation after the toxic treatment caused a further increase of NAD^+^ levels, particularly at the earlier time points following exposure. Yet, 24 h after, the NAD^+^ levels were not higher for NR-treated samples when compared to the respective non-treated samples (Figure 2). As we could observe a positive impact of NR on elevating cellular NAD^+^ levels in the earlier hours following mustard exposure, the effect of NAD^+^ booster was further investigated towards modulating NAD^+^-dependent molecular pathways in mustard-induced genotoxic stress.

**Figure 3:**
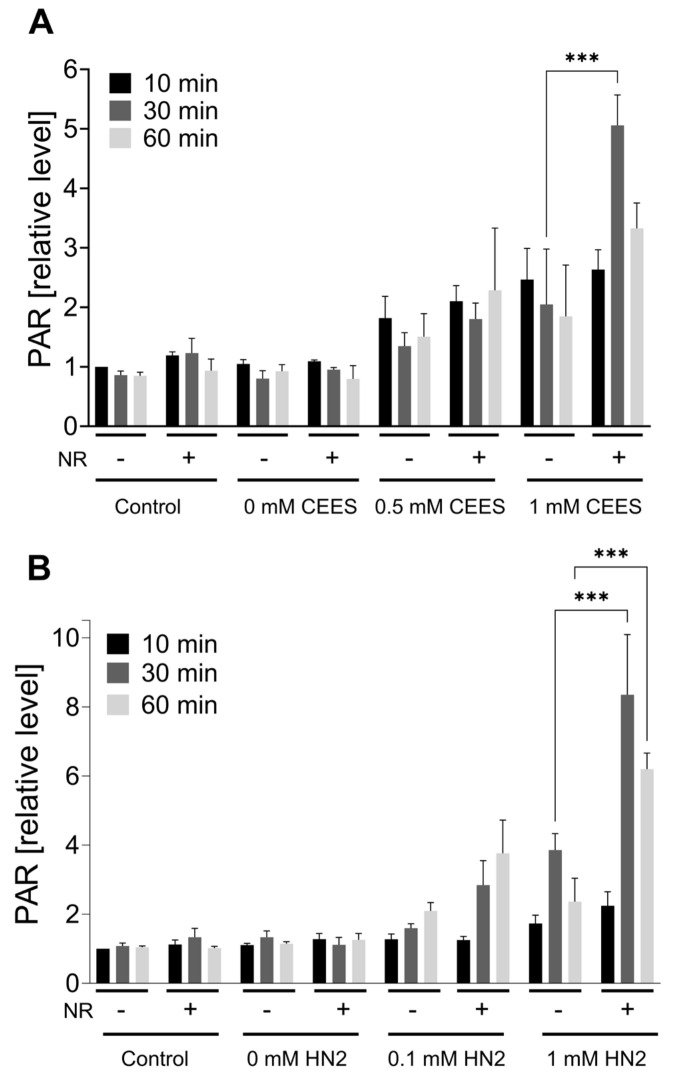
NR elevates PAR levels during genotoxic stress. HaCaT cells were supplemented with 100 μM NR for 3 h (pretreatment) and further treated with CEES ((**A**), n = 3) or HN2 ((**B**), n = 2–3) for 10, 30, or 60 min in PBS (“Control”); “0 mM” refers to solvent control. At the end of each time-point, cells were fixed with ice-cold methanol and stained with anti-PAR antibody (10H) and DNA dye (Hoechst 33342). Images were acquired using epifluorescence microscope and automatically analyzed using KNIME software. Results were normalized to “Control” (10 min) and expressed as mean ± SEM. Results were analyzed by two-way ANOVA and Tukey’s multiple comparison tests. *** *p* < 0.001.

As NAD^+^ depletion upon mustard exposure has been attributed to the PARP1-mediated PAR formation [15,28,31], we tested how NR affects this process in the HaCaT system. The immunofluorescence analysis of nuclear PAR formation (Figure S2) revealed the accumulation of PAR in HaCaT cells upon both CEES (Figure 3A) and HN2 (Figure 3B) exposure in a dose- and time-dependent manner. NR supplementation elevated PAR levels, with the strongest effect observed for the highest doses (an increase of 2.5-fold for 1 mM CEES at 30 min and 2.2-fold for 1 mM HN2 at 30 min, respectively), confirming that NAD^+^ availability is indeed a limiting factor in PARP1 activity upon genotoxic stress [39]. These results provide proof-of-principle for the functional significance of cellular NR supplementation with regards to DNA damage signaling mechanisms. A similar effect of increased polymer formation in the presence of NAD^+^ booster NA has been previously observed upon the X-irradiation of human PBMCs [40] or in rat bone marrow cells treated with the alkylating agent ethylnitrosurea [41]. As elevated PAR levels influence cell signaling in the process of DNA repair, the impact of NR was also investigated in this context. The amount of DNA strand breaks induced by CEES (i.e., which are indicative for DNA repair intermediates) was measured immediately (‘0 h’) or 20 h after exposure, revealing increased levels of DNA breaks upon 4 mM CEES at a later time point (Figure S3A), but with no effect of NR supplementation. Although the previous study showed CEES-induced strand breaks in HaCaT cells at concentrations equal to or higher than 500 μM, that finding might be due to longer (i.e., 60 min) CEES exposure [31]. The absence of the quick induction of strand breaks—despite visible PAR formation—might be related to sensitivity differences or corroborate the fact that DNA strand breaks are not induced by mustards directly but occur during the repair of other DNA damage types, such as adducts [34]. NR had also no impact on the ICL levels detected after HN2 exposure (Figure S3B); HN2 led to a quick, dose-dependent increase in ICL formation, followed by a loss over time (‘24 h’), indicating the repair of these DNA adducts.

**Figure 4:**
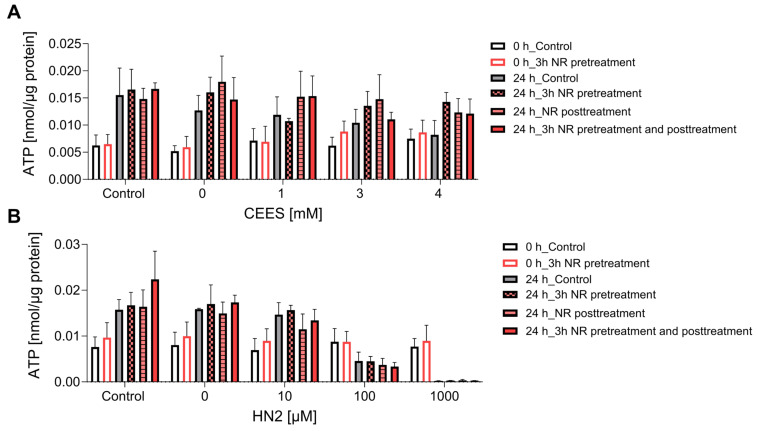
NR does not affect ATP levels during genotoxic stress. HaCaT cells were supplemented with 100 μM NR for 3 h (pretreatment) and subsequently treated with CEES ((**A**), n = 3–4) or HN2 ((**B**), n = 3–4) for 30 min in PBS (“Control”); “0” refers to solvent control. Cells were harvested immediately (0 h) or after 24 h incubation in a fresh growth medium ±100 µM NR (posttreatment). Cellular ATP levels were measured via Cellular ATP Kit HTS and normalized to total protein measured by BCA. Results were expressed as mean ± SEM and analyzed by two-way ANOVA with Tukey’s multiple comparisons test.

ATP is a key molecule in cellular metabolism and signaling, the level of which is dependent on intracellular NAD^+^ content. Thus, NAD^+^ depletion may lead to a decline in ATP, which has been previously observed in HaCaT cells upon SM exposure [15]. In our study, treatment with CEES did not affect ATP levels, as they stayed similar to control levels regardless of the used genotoxicant concentration, or time after exposure (0 h and 24 h) (Figure 4A). HN2 did not affect ATP levels immediately but led to a dose-dependent decrease measured 24 h after exposure (Figure 4B). Accordingly, a decline in ATP levels was previously observed at 6 h or later after keratinocytes were exposed to high concentrations of SM [15,42], indicating that ATP depletion is rather a late event in SM toxicity. NR had no significant effect on ATP levels under the conditions analyzed (Figure 4). Interestingly, an increased cellular ATP content was detected at 24 h when compared to 0 h (Figure 4). As the ATP levels were normalized to the total protein, the increase cannot be explained by cell proliferation. It should be noted that HaCaT cell duplication time is approximately 24 h; thus, at the time point 48 h post-seeding, the culture is likely to be in the log phase. A potentially higher cell division rate may well increase energy demand, thus upregulating ATP levels. This is also consistent with elevated NAD^+^ levels at 24 h (Figure 2A,B).

Similarly, NR had a small effect on cellular response to H_2_O_2_, an oxidizing genotoxic agent and strong PARP1 activator, (Figure S4). H_2_O_2_ induced the formation of PAR polymers (5-fold increase) during 10 min exposure, which was additionally elevated 2.4-fold by NR (Figure S4A,B). The subsequent drop in PAR levels can be attributed to PAR degradation that drives NAD^+^ depletion and, in consequence, cell death. Yet, NR supplementation did not prevent a rapid and massive (80%) cellular NAD^+^ decline after H_2_O_2_ treatment (Figure S4C) after 10 min exposure (0 h), indicating overstressing of the system. At later time points (6 h and 24 h), NAD^+^ was restored to control levels, regardless of NR supplementation. NR did not affect the amount of DNA strand breaks detected immediately after exposure (Figure S4D), although it increased slightly, yet statistically non-significantly, the relative amount of double stranded DNA upon alkaline unwinding at 20 h, suggesting a potential influence on DNA integrity. Since, at this time point, the results are already matching those of control samples, it is not possible to accurately evaluate the impact of NR on DNA damage repair. The H_2_O_2_ treatment also resulted in quick ATP depletion (67%) (Figure S4E), which at 24 h was still decreased (30%, not statistically significant) when compared to the control. NR supplementation slightly mitigated this effect to approximately 20% (Figure S4E).

### 3.2. NR Does Not Affect Short- or Long-Term Cytotoxicity in HaCaT Cells

**Figure 5:**
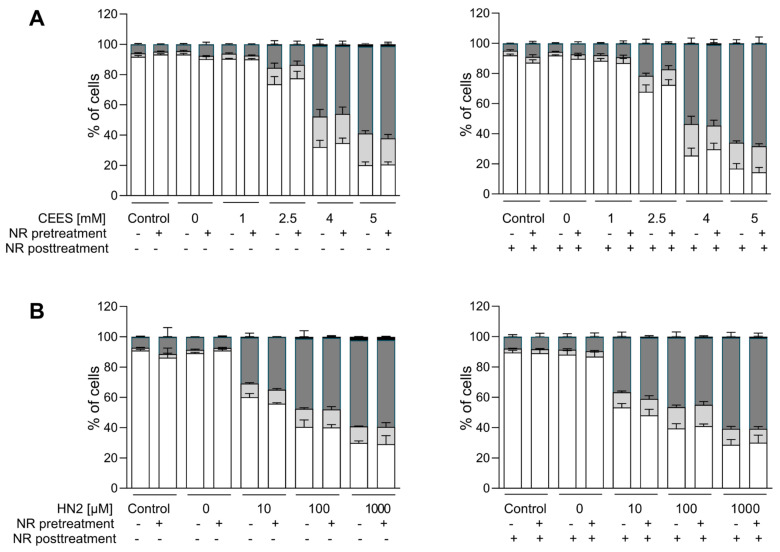
NR does not attenuate cell death induced by genotoxicants. HaCaT cells were supplemented with 100 μM NR for 3 h (pretreatment) and further treated with CEES ((**A**), n = 3) or HN2 ((**B**,) n = 3) for 30 min in PBS (“Control”); “0 mM” refers to solvent control. After exposure, cells were incubated with a fresh growth medium ±100 μM NR (posttreatment) for 24 h. Then, cells were harvested, stained with Annexin V(AV) and propidium iodide (PI), and analyzed via FACS. At least 10,000 cells per sample were measured. Viable cells (AV–/PI–), early apoptotic cells (AV+/PI–), late apoptotic/necrotic cells (AV+/PI+), and dead cells (AV–/PI+) were identified. Results were expressed as mean + SEM and analyzed by two-way ANOVA with Tukey’s multiple comparisons test.

**Figure 6:**
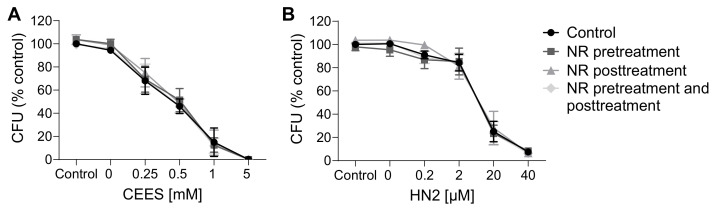
NR does not attenuate cytotoxicity as assessed by clonogenic survival. HaCaT cells were supplemented with 100 μM NR for 3 h (pretreatment) and further treated with CEES ((**A**), n = 3) or HN2 ((**B**), n = 3–4) for 30 min in growth medium (“Control”); “0 mM” refers to solvent control. After treatment, cells were reseeded 1000 cells/well in technical triplicates and allowed to grow for 7 days in fresh growth medium ±100 µM NR (posttreatment). Next, colonies were stained with crystal violet and counted using OpenCFU software. For each experiment, an average from technical replicates of the colony-forming unit (CFU) was calculated and normalized to the control (growth medium without NR). Results were expressed as mean ± SEM and analyzed by two-way ANOVA with Tukey’s multiple comparison test.

In agreement with results reported in the previous section, NR had also no effect on the cytotoxicity of the genotoxicants tested. Both CEES (Figure 5A) and HN2 (Figure 5B) treatments led to a dose-dependent decrease in the number of viable cells, in parallel with the increasing number of dying cells, in particular those exhibiting late apoptosis and necrosis marker (Annexin V and PI-positive). NR supplementation neither improved cell viability nor did it affect the type of cell death. This is in line with the lack of significant NR effects on cellular NAD^+^ levels (Figure 2) or ATP levels (Figure 4) at 24 h. Besides short-term toxicity, the effect of NR on the cellular potential of clonogenicity was evaluated (Figure 6). Clonogenic survival is a long-term in vitro toxicity assay, which, in addition to cell death, takes into account cell adhesion and proliferation, thus showing much higher sensitivity regarding the detection of toxic effects. In fact, the test revealed harmful effects of concentrations much lower than those identified as cytotoxic in the short-term assay, especially for CEES. Again, NR supplementation had no impact on these outcomes. Likewise, NR had no significant effect on clonogenic survival (Figure S5A) or cell death (Figure S5B–F) following H_2_O_2_ exposure; although, interestingly, NR supplementation post-treatment seemed to slightly potentiate cell death, with the increase in late apoptotic and necrotic cells (from 23% to 34%, not significant).

### 3.3. NAD^+^ Depletion Sensitizes Cells to Genotoxic Stress in a Compound-Specific Manner

**Figure 7:**
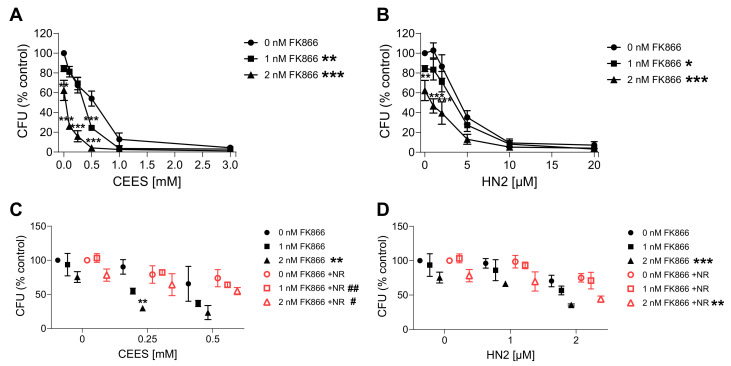
NR attenuates the sensitizing effect of FK866 on genotoxic stress. HaCaT cells were treated with FK866 in 0.5% DMSO (0 nM FK866) for 24 h and then exposed to CEES ((**A**), n = 2–3) or HN2 ((**B**), n = 3) for 30 min in growth medium; “0 mM” refers to solvent control. Subsequently, cells were reseeded 1000 cells per well in technical triplicates and incubated in fresh growth medium with or without FK866 for 7 days. Then, colonies were stained and counted. For each experiment, an average from technical replicates of the colony-forming unit (CFU) was calculated and normalized to the control (0 nM FK866). (**C**,**D**) HaCaT cells were treated with CEES ((**C**), n = 3) or HN2 ((**D**), n = 3); additionally, FK866 and 100 µM NR were added to the culture as described in Materials and Methods, and samples were analyzed similar to that in (A,B). Results were expressed as mean ± SEM and analyzed by two-way ANOVA with Tukey’s multiple comparison test. * *p* < 0.05, ** *p* < 0.01, *** *p* < 0.001 vs. “0 nM FK866; # *p* < 0.05, ## *p* < 0.01 vs. respective samples without NR.

**Figure 8:**
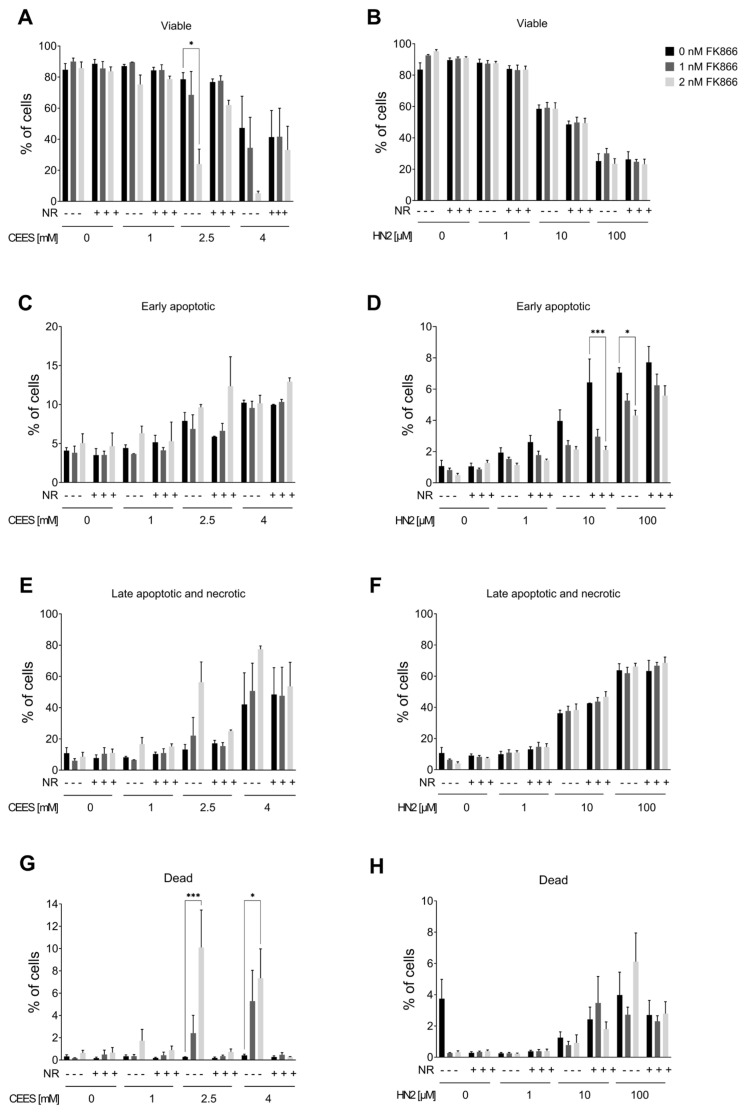
NR attenuates the sensitizing effect of FK866 on cell death. HaCaT cells were treated with FK866 in 0.5% DMSO (0 nM FK866) for 24 h; additionally, 100 µM NR was added to the culture as described in Materials and Methods. Next, cells were exposed to CEES ((**A**,**C**,**E**,**G**); n = 3) or HN2 ((**B**,**D**,**F**,**H**); n = 3–4) for 30 min in PBS; “0” refers to solvent control. After exposure, cells were washed, fresh growth medium with or without FK866 and with or without NR was applied, and cells were incubated for 24 h. Then, cells were harvested, stained with Annexin V(AV) and propidium iodide (PI), and analyzed via FACS. At least 10,000 cells per sample were measured. Viable cells (AV–/PI–), early apoptotic cells (AV+/PI–), late apoptotic/necrotic cells (AV+/PI+), and dead cells (AV–/PI+) were identified. Results were expressed as mean + SEM and analyzed by two-way ANOVA with Tukey’s multiple comparisons test. * *p* < 0.05, *** *p* < 0.001.

Since the elevation of NAD^+^ levels poorly attenuated the toxicity of mustards, as shown in the present and previous studies [43,44], yet NAD^+^ in general plays crucial roles in cellular physiology [19], the hypothesis that NAD^+^ participates in mustard-induced mechanisms of toxicity was further examined by experimentally depleting NAD^+^ levels. To address this view, prior to genotoxicant exposure, we decreased cellular NAD^+^ levels via FK866 treatment, which is a specific inhibitor of NAMPT, the key enzyme in the salvage pathway, which converts NAM to NMN (Figure 1A) [26]. We tested two FK866 concentrations, i.e., 1 nM and 2 nM. While these concentrations led to relatively low cytotoxicity in the AlamarBlue assay (88% and 69% cell viability, respectively) (Figure S6A), significant depletion of cellular NAD^+^ levels was observed (42% and 0% of control levels, respectively) (Figure S6B) after two consecutive applications (24 + 24 h) (N.B., this represents the treatment for 24 h before and immediately after genotoxicant exposure). FK866 administration impaired the clonogenic survival of HaCaT cells exposed to SM analogues in a concentration-dependent manner (Figure 7A,B). Already, 1 nM FK866 sensitized HaCaT cells, with the effect on CEES long-term toxicity being more pronounced (Figure 7A) in comparison to HN2 (Figure 7B) or H_2_O_2_ (Figure S5C). Consequently, 2 nM FK866 produced a stronger effect, but it was toxic itself and significantly reduced the number of cell colonies in control samples. Of note, the FK866 effect was abrogated by NR supplementation, which rescued NAD^+^ decrease (Figure S6C). NR attenuated the FK866 effect on clonogenic survival after CEES exposure (Figure 7C). For example, for 0.25 mM CEES, NR increased clonogenicity from 55% to 82% for 1 nM FK866 and from 30% to 64% for 2 mM FK866, respectively. In the case of HN2, the NR effect was also observed but less pronounced (Figure 7D). Moreover, NR supplementation affected the FK866 impact on mustard-induced cell death (Figure 8A–H). The treatment with 2 nM FK866 significantly decreased the number of viable cells (Figure 8A) and increased the percentage of dying cells upon CEES exposure (Figure 8E,G), while NR supplementation attenuated these effects. For example, for 2.5 mM CEES, 2 nM FK866 reduced the number of viable cells from 79% to 24%, whereas NR supplementation resulted in 77% and 62%, respectively. Interestingly, such effects were not observed for exposure to HN2 (Figure 8B,D,F,H) or H_2_O_2_ (Figure S5D–G), implying distinct modes of cell death. In turn, FK866 showed a trend toward decreasing the number of apoptotic cells in the cases of HN2 (Figure 8D) and H_2_O_2_ (Figure S5E), but NR supplementation had no clear effect on these outcomes. It is noteworthy that 2 nM FK866 had no significant effect on cell viability in this short-term toxicity assay, in comparison to the clonogenic survival assay (Figure 7A–D). Together, these data demonstrate that maintaining cellular NAD^+^ levels is indeed important for cell survival upon genotoxic stress exposure, but with compound-specific sensitivity.

### 3.4. NR Attenuates THP-1 Susceptibility to Mustards

**Figure 9:**
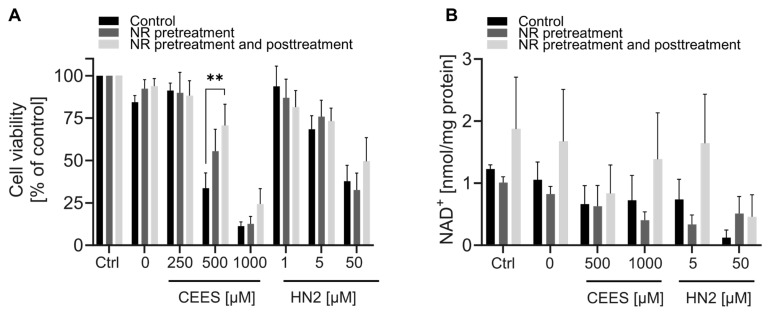
Effects of NR supplementation in THP-1 cells exposed to CEES or HN2. (**A**) THP-1 cells were supplemented with 200 µM NR for 4 h before treatment with CEES or HN2 in growth medium (“Ctrl”) for 30 min. The “0” refers to solvent control. After exposure, cells were incubated with fresh growth medium with or without 200 µM NR for 20 h, and cell viability was measured with alamarBlue assay. Results were normalized to the control “Ctrl” and expressed as mean ± SEM (n = 3–4). (**B**) After treatment, similarly to that in (**A**), THP-1 cells were incubated for 24 h, and cellular NAD^+^ was extracted. NAD^+^ levels were measured via enzymatic cycling assay and normalized to the total protein level measured by BCA (n = 2–3). Data were analyzed by two-way ANOVA with Tukey’s multiple comparisons test. ** *p* < 0.01.

In addition, we analyzed the NR effect in the monocytic-like cell line THP-1. These cells exhibited similar responses to mustards, as a concentration-dependent decrease of cellular NAD^+^ levels was observed 24 h after exposure to CEES or HN2 (Figure S7A). THP-1 cells also showed a dose-dependent increase in cellular NAD^+^ levels due to NR exposure (Figure S7B): treatment with 50–200 µM NR for 4 h elevated NAD^+^ levels significantly, indicating that NR was efficiently metabolized to NAD^+^. The supplementation resulted in a stable increase in cellular NAD^+^ levels. When NR-containing growth medium was replaced with NR-free medium, the NAD^+^ levels remained elevated for up to 5 h after exchange (Figure S7B). As the 4 h treatment with 200 µM NR resulted in the highest increase of NAD^+^ levels, this protocol was further implemented to study the NR effect on THP-1 susceptibility to mustards (Figure 9). NR showed protective effects on cell viability upon mustard exposure. The effect was significant for 500 µM CEES, and a similar trend was also evident for 1000 µM CEES (Figure 9A). For HN2-treated cells, viability was not significantly affected by NR; although, at a concentration of 50 µM HN2, NR pre- and posttreatment showed a slight (non-significant) increase in cell survival, as compared to the control (Figure 9A). Moreover, NR supplementation showed elevated (statistically non-significant) cellular NAD^+^ levels upon mustard exposure, especially after NR pre- and posttreatments (Figure 9B).

## 4. Discussion

The present study provides a comparative examination of the role of NAD^+^ in the toxicity of two sulfur mustard analogues, CEES and HN2, in human keratinocytes HaCaT and monocytes THP-1. The main objective of this study was to assess the effects of NAD^+^ supplementation on the molecular pathways impaired by DNA-damaging mustard exposure. The systematic comparison of NAD^+^ supplements revealed that NR is the most effective NAD^+^ booster in HaCaT cells (Figure 1B). Other compounds were less efficient; for example, NAM showed a very low increase (Figure 1C), which was somewhat unexpected, given the frequent application of this compound in previous HaCaT studies [45,46]. To our knowledge, the NAM influence on NAD^+^ levels in HaCaT cells has not been reported so far, despite multiple studies investigating other NAM supplementation effects on several cellular endpoints [45,46]. The low impact of NAM supplementation might be due to the culture conditions, as the DMEM medium used in this study already contains 33 µM NAM. That might explain the lack of effectiveness of additional NAM supplementation, as well as a slight (not significant) increase in NAD^+^ levels over time following media change in control samples (Figure 1). On the other hand, the lack of NAM response might be related to its possible inhibitory effects on NAD^+^-consuming enzymes, such as ARTDs and SIRTs [20]. Studies in other systems indicate NAM effectiveness, e.g., in human keratinocytes, 1 mM NAM resulted in an NAD^+^ increase of approximately 30% or 60% after 4 h or 24 h exposure, respectively [43]. Another surprising result was the lower efficiency of NMN (Figure 1D) when compared to NR (Figure 1B), given that NMN is a direct precursor to NAD^+^, whereas NR needs to be beforehand transformed into NMN by NRK (Figure 1A). The cause is not clear, but compound stability or limited transport across cellular membranes may play a role, as NMN is a larger molecule than NR and contains negatively charged phosphate. The cellular NMN uptake is under discussion—the recently proposed transporter remains controversial [47,48]—and the leading hypothesis comprises the dephosphorylation of NMN into NR in order to enter the cells [49]. To our knowledge, this is the first study investigating these compounds in HaCaT cells. Thus, in light of complex and not fully described pharmacokinetics [50], the scrutinized characterization of specific NAD^+^ supplements prior to further tests in new systems is strongly recommended.

The fact that NR produced the highest increase in cellular NAD^+^ levels is not surprising given numerous reports on its exceptionally high bioavailability in humans [24,25,51]. However, what clearly emerges from our study is the limited boosting of the NAD^+^ pool. NR supplementation increased the NAD^+^ levels in a dose-dependent manner progressively during the first hours of exposure (up to 6 h). Yet, the 24 h incubation did not result in a similar increase (Figure 1 and Figure 2), suggesting either an early exhaustion of the NR pool, feedback inhibition of NAD^+^ synthesis, or overactivation of NAD^+^-consuming enzymes. One human study showed that participants receiving daily a high dose of NR showed a relative decline of NAD^+^ levels with time (30 vs. 60 days), which authors interpreted as the result of the induction of NAD^+^ degrading enzymes [51]. A potential way to prevent NAD^+^ decline with time would be frequent NR re-application. However, one needs to be careful, as the biological effects of prolonged NAD^+^ boosting are unclear and might possibly cause adverse effects on cellular homeostasis [52]. For example, NR has been shown to decrease endogenous antioxidant activity and impair redox homeostasis in young rats [53]. Moreover, continuous NAD^+^ elevation may have particularly detrimental effects on systems where intracellular NAD^+^ levels normally oscillate according to the circadian rhythm [54]. 

Nevertheless, the initially significant elevation of cellular NAD^+^ levels provided a window of opportunity to investigate possible protective effects of NR against early events of genotoxic stress induced by mono- and bi-functional mustard agents. Hence, in our experimental conditions, NR, despite significantly elevating NAD^+^ (Figure 2) and PARylation response (Figure 3), did not afford protection in regard to cytotoxicity assays (Figure 5 and Figure 6) or other molecular endpoints (Figure 4 and Figure S3). This is in line with numerous earlier studies demonstrating insufficient protection of NAD^+^ precursors (NA and NAM) in mustard toxicity, as reviewed elsewhere [19]. The lack of a positive effect of NR might be partially explained by the fact that despite a NAD^+^ increase within the first hours of NR supplementation, at 24 h, NAD^+^ matched the control levels (Figure 2). This indicates that achieving elevated NAD^+^ levels in the moment of initial stress events is not sufficient to attenuate the genotoxic and cytotoxic effects of SM analogues. Therefore, implementing continuous NAD^+^ elevation for extended time periods should be further investigated, keeping in mind the risks, mentioned above, associated with long-term NAD^+^ supplementation.

An alternative explanation considers the secondary role of NAD^+^ in the cytotoxicity of alkylating agents, which is reflected by the lack of long-term protective effects of NAD^+^ supplementation [43,55,56,57] or conflicting outcomes of PARP inhibition [15,28]. This hypothesis proposes that mustards inhibit glycolysis directly; thus, NAD^+^ depletion plays rather a minor role, together with other factors. It is supported by observations that ATP depletion occurs much later than reduced glucose utilization [43]. However, in a previous study, the application of PARP inhibitors revealed the functional role of NAD^+^-dependent PAR synthesis in genotoxic stress response in HaCaT [28]. In the present study, we examined the role of NAD^+^ per se, with the use of NAD^+^ synthesis inhibitor FK866 (Figure 7). The depletion of cellular NAD^+^ levels by FK866 showed a substantial sensitization of HaCaT to alkylating agents. Of note, the effect was more pronounced for CEES than HN2 treatment, corroborating substance-specificity and underscoring the different cellular mechanisms of toxicity for these compounds in HaCaT [28,58]. In this study, besides the higher toxicity of HN2 than CEES, other differences encompass a less apparent NAD^+^ (Figure 2) and ATP (Figure 4) decline after CEES than HN2 exposure for concentrations displaying similar cytotoxicity (4 mM CEES and 100 µM HN2) (Figure 5). The higher sensitization of CEES than HN2 towards NAD^+^-dependent mechanisms has also been observed in the previous study. PARP inhibition sensitized HaCaT cells mostly to SM and CEES, and to a lesser extent to HN2, as demonstrated by clonogenic survival, cell proliferation, or micronucleus formation assays [28]. Together, these results indicate that the HN2 toxicity mechanism is fairly independent of NAD^+^ levels or PARP activity.

Similarly, maintaining NAD^+^ levels played a minor role in H_2_O_2_-induced cell death (Figure S5C–G); although, early PARP1 activation seemed to influence H_2_O_2_ toxicity, as PARP inhibition prevented a massive NAD^+^ depletion and cell death following H_2_O_2_ treatment, which is in line with rescuing NAD^+^ levels and consequently increasing cell survival [28]. Moreover, for both HN2 (Figure 7 and Figure 8) and H_2_O_2_ (Figure S5C–G), NR supplementation had little influence on FK866-dependent effects. By contrast, CEES-treated cells exhibited the highest sensitivity to NAD^+^ depletion and PARP inhibition, indicating that both molecules contribute to cell survival following half mustard exposure. NAD^+^ deprivation sensitized cells to CEES particularly to necrosis, rather than apoptosis, although a slight increase in apoptotic cells was also observed (Figure 8C). Overall, the FK866-induced increase in cell death following CEES exposure was largely rescued by NR supplementation, although with distinct effects (NR caused a slight increase in the apoptotic cells (Figure 8C) but a decrease in necrotic and dead cells (Figure 8E,G)). This effect is consistent with NAD^+^ being essential for the execution of apoptosis, whereas NAD^+^ deprivation leads to necrosis. An earlier study demonstrated that PARP inhibition (by preserving NAD^+^ and ATP pools) reduced the induction of necrosis and ensured the appropriate execution of caspase-mediated apoptosis in HaCaT cells after SM treatment [15]. Here, we showed that, following CEES exposure, initially, more cells were in early apoptosis than for HN2; moreover, FK866 treatment did not have a clear effect, indicating that there may be an alternative mechanism other than that decreased NAD^+^ levels and subsequent energy crisis trigger apoptosis in those cells. For example, CEES has been shown to induce apoptosis via Erk1/2 mitogen–activated protein kinases (MAPKs) and phosphatidylinositol-3-kinase (PI3K/Akt) signaling cascades in mouse keratinocytes JB6 [59,60]. Additionally, the phosphorylation of ataxia-telangiectasia mutated ATM and ataxia telangiectasia and Rad3 related (ATR) have been identified as early events of CEES cytotoxicity in HaCaT and JB6 [61].

Unlike in HaCaT, in THP-1, we observed a protective NR effect on cell viability, particularly upon CEES exposure (Figure 9A). These data further support the observations in HaCaT (Figure 7 and Figure 8) that maintaining cellular NAD^+^ levels might be particularly beneficial in the case of CEES-induced toxicity. Additionally, these findings demonstrate that NR supplementation attenuates genotoxic stress in a system-specific manner. The reason behind the differential response of HaCaT and THP-1 is not clear, but data obtained suggest the following explanation: THP-1 cells seem to have lower basal NAD^+^ content, as the untreated cells exhibited on average 1.48 nmol/mg of protein (Figure S7A), whereas for HaCaT it was 3.2 nmol/mg of protein (Figure 2A). Lower basal NAD^+^ levels might provide room for further elevation via supplementation, whereas a highly saturated system, such as HaCaT, might be less susceptible to further NAD^+^ elevation. Additionally, the NAD^+^ metabolism seems to be slower in THP-1 cells where NAD^+^ levels remained elevated for up to 5 h after NR removal from the growth medium (Figure S7B), whereas in HaCaT, the NAD^+^ levels at 5 h were not different from the untreated control (Figure S1). Thus, longer-lasting NAD^+^ elevation in THP-1, following mustard exposure (Figure 9B), might contribute to the higher resistance of cells (Figure 9A). These results corroborate previous studies, showing beneficial effects of NR supplementation in disease and ageing associated with lower basal NAD^+^ levels, but not in young healthy animals/individuals exhibiting higher NAD^+^ levels [20,62,63,64].

## 5. Conclusions

In conclusion, this study shows that elevating cellular NAD^+^ levels with NR provides system-specific protection against genotoxicity induced by the sulfur mustard analogue CEES, suggesting that NR is a promising intervention for certain types of cells exposed to mustards. NR showed positive outcomes particularly in the conditions of initially lower NAD^+^ levels, providing evidence of the compound-specific functional role of NAD^+^ in the mustards’ toxicity pathomechanism. Apart from new insight into the status of NAD^+^ in mustard toxicity, our study suggests the particularly beneficial therapeutic application of NR supplementation in the context of reduced basal NAD^+^ levels, which is the phenomenon associated with certain diseases and ageing [20]. Further studies in appropriate in vivo and ex vivo models are needed to investigate these effect.

## Data Availability

All relevant data are included in the main manuscript or the Supplementary Materials. Raw data are available upon reasonable request from the corresponding authors.

## Supplementary Materials

The following supporting information can be downloaded at: https://www.mdpi.com/article/10.3390/cells12192396/s1, Figure S1: NR-dependent cellular NAD+ increase. HaCaT cells were exposed to NR for 3 h, next the growth medium was exchanged for fresh medium without NR, and cells were harvested at indicated time points. Cellular NAD+ levels were measured via enzymatic cycling assay and data were normalized to the control “0 µM” at 0 h. The results were expressed as mean ±SEM and analyzed by two-way ANOVA with Tukey’s multiple comparisons test (n = 4). * *p* < 0.05, ** *p* < 0.01, *** *p* < 0.001, Figure S2: NR elevates PAR levels during genotoxic stress. HaCaT cells were supplemented with 100 μM NR for 3 h and further treated with CEES or HN2 for 10, 30, or 60 min in PBS; “0 mM” refers to solvent control. At the end of each time point cells were fixed with ice-cold methanol, stained with anti-PAR antibody (10H) and DNA dye (Hoechst) and the signal was captured using an epifluorescence microscope. Scale bar indicates 100 µm, Figure S3: NR does not affect the formation and repair of DNA damage induced by alkylating agents. HaCaT cells were supplemented with 100 μM NR for 3 h and further treated with CEES (A, n = 3), or HN2 (B, n = 4) for 30 min in PBS. “0” refers to solvent control. Next cells were washed and incubated with fresh growth medium ±100 µM NR for 20 h (A) or 24 h (B), or harvested immediately (0 h). Induction of DNA strand breaks, which is inversely proportional to the relative levels of double-stranded DNA (dsDNA) remaining after alkaline unwinding, by CEES was measured via fluorimetric detection of alkaline DNA unwinding (FADU) assay (A), whereas induction of inter-strand crosslinks (ICLs) by HN2 was measured with reversed automated fluorimetric detection of alkaline DNA unwinding (rFADU) assay. Results were expressed as mean +SEM and analyzed by two-way ANOVA with Tukey’s multiple comparisons test. *** *p* < 0.001, Figure S4: Effects of NR on H2O2 response in HaCaT. A: HaCaT cells were supplemented with 100 μM NR for 3 h and further treated with 100 µM H2O2 for 10, 30, or 60 min in PBS (“Control”). At the end of each treatment cells were fixed with ice-cold methanol, stained with anti-PAR antibody (10H) and DNA dye (Hoechst), and the signal was captured using an epifluorescence microscope. Scale bars indicate 100 µm, B: Images were automatically analyzed using KNIME software, and results were normalized to “Control” (10 min) (n = 3). C: HaCaT cells were supplemented with 100 μM NR for 3 h and further treated with 100 µM H2O2 for 10 min. Next, cells were incubated in fresh growth medium with or without NR (up to 24 h) or harvested immediately (0 h). The NAD+ levels were measured via enzymatic cycling assay, normalized to the total protein level measured by BCA, and expressed as fold change of “Control” at 0 h (n = 2–3). D: Formation of DNA strand breaks was measured via fluorimetric detection of alkaline DNA unwinding (FADU) assay (n = 3). E: ATP levels were measured via Cellular ATP Kit HTS and normalized to the total protein measured by BCA (n = 3–4). Results were expressed as mean +SEM and analyzed by two-way ANOVA with Tukey’s multiple comparisons test. * *p* < 0.05, *** *p* < 0.001, Figure S5: Effects of NR on H2O2 (geno)toxicity. HaCaT cells were supplemented with 100 µM NR for 3 h and further treated with H2O2 for 10 min. A: After treatment, cells were reseeded 1000 cells per well in technical triplicates and incubated in fresh growth medium ±NR for 7 days. Then colonies were stained and counted. For each experiment, an average from technical replicates of the colony-forming unit (CFU) was calculated and normalized to “Control” (0 µM H2O2) (n = 3). B: After treatment cells were incubated in fresh growth medium with or without NR for 24 h and cell death was analyzed via flow cytometry as described in Materials and methods (n = 6), C: Cells were treated and analyzed like in (A), additionally FK866 was added to the culture, as described in Materials and methods (n = 3); D-G: Cells were treated and analyzed via FACS like in (B), additionally FK866 was added to the culture, as described in Materials and methods (n = 3–4). Results were expressed as mean ±SEM and analyzed by two-way ANOVA with Tukey’s multiple comparisons test. * *p* < 0.05, Figure S6: The effects of FK866 on HaCaT cells. HaCaT cells were treated with FK866 in 0.5% DMSO (0 nM FK866) in growth medium for 24 h or 48 h, additionally, the repeated exposure treatment (24 + 24 h) was applied, where fresh growth medium with FK866 was replaced after 24 h and cells were incubated for additional 24 h. A: Cell viability was measured via alamarBlue assay and results were normalized to “0 nM” (n = 3). B: Cellular NAD+ levels were measured via enzymatic cycling assay and normalized to the total protein level measured by BCA (n = 3). C: HaCaT cells were treated with FK866 for 24 h and 24 + 24 h as in (A), additionally 100 µM NR was applied either 3 h before cell harvesting during 24 h FK866 exposure, or with the new growth medium (3 + 24 h) for 24 + 24 h FK866 exposure. Cellular NAD+ levels were measured via enzymatic cycling assay and normalized to the total protein level measured by BCA (n=3). Results were expressed as mean ±SEM and analyzed by two-way ANOVA with Tukey’s multiple comparisons test. ** *p* < 0.01, *** *p* < 0.001, Figure S7: NAD+ levels upon mustards and NR treatments in THP-1 cells. A: THP-1 cells were exposed to CEES or HN2 in growth medium (“Control”) for 30 min; “0” refers to solvent control. Next, cells were washed and incubated with fresh growth medium for 24 h, when cells were harvested for NAD+ extraction (n = 2). B: THP-1 cells were exposed to NR for 4 h, then the growth medium was replaced with fresh medium without NR, and cells were harvested up to 5 h later and cellular NAD+ was extracted (n = 4). The NAD+ levels were measured via enzymatic cycling assay and normalized to the total protein level measured via BCA. The results were expressed as mean ±SEM and analyzed by two-way ANOVA with Tukey’s multiple comparisons test. *** *p* < 0.001.

## References

[1] Mangerich A., Esser C. (2014). Chemical warfare in the First World War: Reflections 100 years later. Arch. Toxicol..

[2] Panahi Y., Abdolghaffari A.H., Sahebkar A. (2018). A review on symptoms, treatments protocols, and proteomic profile in sulfur mustard-exposed victims. J. Cell. Biochem..

[3] Rahmani S., Abdollahi M. (2017). Novel treatment opportunities for sulfur mustard-related cancers: Genetic and epigenetic perspectives. Arch. Toxicol..

[4] Etemad L., Moshiri M., Balali-Mood M. (2019). Advances in treatment of acute sulfur mustard poisoning—A critical review. Crit. Rev. Toxicol..

[5] Neidle S., Thurston D.E. (2005). Chemical approaches to the discovery and development of cancer therapies. Nat. Rev. Cancer.

[6] Ludlum D.B., Austin-Ritchie P., Hagopian M., Niu T.-Q., Yu D. (1994). Detection of sulfur mustard-induced DNA modifications. Chem. Biol. Interact..

[7] Zubel T., Hochgesand S., John H., Steinritz D., Schmidt A., Bürkle A., Mangerich A. (2019). A mass spectrometric platform for the quantitation of sulfur mustard-induced nucleic acid adducts as mechanistically relevant biomarkers of exposure. Arch. Toxicol..

[8] Kehe K., Balszuweit F., Steinritz D., Thiermann H. (2009). Molecular toxicology of sulfur mustard-induced cutaneous inflammation and blistering. Toxicology.

[9] Powell K.L., Boulware S., Thames H., Vasquez K.M., MacLeod M.C. (2010). 2,6-Dithiopurine blocks toxicity and mutagenesis in human skin cells exposed to sulfur mustard analogues, 2-chloroethyl ethyl sulfide and 2-chloroethyl methyl sulfide. Chem. Res. Toxicol..

[10] Ray Chaudhuri A., Nussenzweig A. (2017). The multifaceted roles of PARP1 in DNA repair and chromatin remodelling. Nat. Rev. Mol. Cell Biol..

[11] Papirmeister B., Gross C.L., Meier H.L., Petrali J.P., Johnson J.B. (1985). Molecular basis for mustard-induced vesication. Fundam. Appl. Toxicol..

[12] Bhute V.J., Palecek S.P. (2015). Metabolic responses induced by DNA damage and poly (ADP-ribose) polymerase (PARP) inhibition in MCF-7 cells. Metabolomics Off. J. Metabolomic Soc..

[13] Lu P., Hogan-Cann A.D., Kamboj A., Roy Chowdhury S.K., Aghanoori M.-R., Fernyhough P., Anderson C.M. (2019). Poly(ADP-ribose) polymerase-1 inhibits mitochondrial respiration by suppressing PGC-1α activity in neurons. Neuropharmacology.

[14] Tateishi K., Higuchi F., Miller J.J., Koerner M.V.A., Lelic N., Shankar G.M., Tanaka S., Fisher D.E., Batchelor T.T., Iafrate A.J. (2017). The Alkylating Chemotherapeutic Temozolomide Induces Metabolic Stress in IDH1-Mutant Cancers and Potentiates NAD(+) Depletion-Mediated Cytotoxicity. Cancer Res..

[15] Kehe K., Raithel K., Kreppel H., Jochum M., Worek F., Thiermann H. (2008). Inhibition of poly(ADP-ribose) polymerase (PARP) influences the mode of sulfur mustard (SM)-induced cell death in HaCaT cells. Arch. Toxicol..

[16] Beigi Harchegani A., Khor A., Tahmasbpour E., Ghatrehsamani M., Bakhtiari Kaboutaraki H., Shahriary A. (2019). Role of oxidative stress and antioxidant therapy in acute and chronic phases of sulfur mustard injuries: A review. Cutan. Ocul. Toxicol..

[17] Stenger B., Popp T., John H., Siegert M., Tsoutsoulopoulos A., Schmidt A., Mückter H., Gudermann T., Thiermann H., Steinritz D. (2017). N-Acetyl-l-cysteine inhibits sulfur mustard-induced and TRPA1-dependent calcium influx. Arch. Toxicol..

[18] Borna H., Hosseini Qale Noe S.H., Harchegani A.B., Talatappe N.R., Ghatrehsamani M., Ghanei M., Shahriary A. (2019). A review on proteomics analysis to reveal biological pathways and predictive proteins in sulfur mustard exposed patients: Roles of inflammation and oxidative stress. Inhal. Toxicol..

[19] Ruszkiewicz J.A., Burkle A., Mangerich A. (2020). NAD(+) in sulfur mustard toxicity. Toxicol. Lett..

[20] Covarrubias A.J., Perrone R., Grozio A., Verdin E. (2021). NAD(+) metabolism and its roles in cellular processes during ageing. Nat. Rev. Mol. Cell Biol..

[21] Katsyuba E., Romani M., Hofer D., Auwerx J. (2020). NAD+ homeostasis in health and disease. Nat. Metab..

[22] Mateuszuk Ł., Campagna R., Kutryb-Zając B., Kuś K., Słominska E.M., Smolenski R.T., Chlopicki S. (2020). Reversal of endothelial dysfunction by nicotinamide mononucleotide via extracellular conversion to nicotinamide riboside. Biochem. Pharmacol..

[23] Neelakantan H., Vance V., Wetzel M.D., Wang H.-Y.L., McHardy S.F., Finnerty C.C., Hommel J.D., Watowich S.J. (2018). Selective and membrane-permeable small molecule inhibitors of nicotinamide N-methyltransferase reverse high fat diet-induced obesity in mice. Biochem. Pharmacol..

[24] Conze D., Brenner C., Kruger C.L. (2019). Safety and Metabolism of Long-term Administration of NIAGEN (Nicotinamide Riboside Chloride) in a Randomized, Double-Blind, Placebo-controlled Clinical Trial of Healthy Overweight Adults. Sci. Rep..

[25] Trammell S.A., Schmidt M.S., Weidemann B.J., Redpath P., Jaksch F., Dellinger R.W., Li Z., Abel E.D., Migaud M.E., Brenner C. (2016). Nicotinamide riboside is uniquely and orally bioavailable in mice and humans. Nat. Commun..

[26] Hasmann M., Schemainda I. (2003). FK866, a highly specific noncompetitive inhibitor of nicotinamide phosphoribosyltransferase, represents a novel mechanism for induction of tumor cell apoptosis. Cancer Res..

[27] Wang Q.Q., Begum R.A., Day V.W., Bowman-James K. (2012). Sulfur, oxygen, and nitrogen mustards: Stability and reactivity. Org. Biomol. Chem..

[28] Mangerich A., Debiak M., Birtel M., Ponath V., Balszuweit F., Lex K., Martello R., Burckhardt-Boer W., Strobelt R., Siegert M. (2016). Sulfur and nitrogen mustards induce characteristic poly(ADP-ribosyl)ation responses in HaCaT keratinocytes with distinctive cellular consequences. Toxicol. Lett..

[29] Boukamp P., Petrussevska R.T., Breitkreutz D., Hornung J., Markham A., Fusenig N.E. (1988). Normal keratinization in a spontaneously immortalized aneuploid human keratinocyte cell line. J. Cell Biol..

[30] Tsuchiya S., Yamabe M., Yamaguchi Y., Kobayashi Y., Konno T., Tada K. (1980). Establishment and characterization of a human acute monocytic leukemia cell line (THP-1). Int. J. Cancer.

[31] Debiak M., Lex K., Ponath V., Burckhardt-Boer W., Thiermann H., Steinritz D., Schmidt A., Mangerich A., Burkle A. (2016). Immunochemical analysis of poly(ADP-ribosyl)ation in HaCaT keratinocytes induced by the mono-alkylating agent 2-chloroethyl ethyl sulfide (CEES): Impact of experimental conditions. Toxicol. Lett..

[32] Jacobson E.L., Jacobson M.K. (1976). Pyridine nucleotide levels as a function of growth in normal and transformed 3T3 cells. Arch. Biochem. Biophys..

[33] Geissmann Q. (2013). OpenCFU, a new free and open-source software to count cell colonies and other circular objects. PLoS ONE.

[34] Debiak M., Panas A., Steinritz D., Kehe K., Burkle A. (2011). High-throughput analysis of DNA interstrand crosslinks in human peripheral blood mononuclear cells by automated reverse FADU assay. Toxicology.

[35] Moreno-Villanueva M., Pfeiffer R., Sindlinger T., Leake A., Muller M., Kirkwood T.B., Burkle A. (2009). A modified and automated version of the ‘Fluorimetric Detection of Alkaline DNA Unwinding’ method to quantify formation and repair of DNA strand breaks. BMC Biotechnol..

[36] Kawamitsu H., Hoshino H., Okada H., Miwa M., Momoi H., Sugimura T. (1984). Monoclonal antibodies to poly(adenosine diphosphate ribose) recognize different structures. Biochemistry.

[37] Rank L., Veith S., Gwosch E.C., Demgenski J., Ganz M., Jongmans M.C., Vogel C., Fischbach A., Buerger S., Fischer J.M. (2016). Analyzing structure-function relationships of artificial and cancer-associated PARP1 variants by reconstituting TALEN-generated HeLa PARP1 knock-out cells. Nucleic Acids Res..

[38] Martens C.R., Denman B.A., Mazzo M.R., Armstrong M.L., Reisdorph N., McQueen M.B., Chonchol M., Seals D.R. (2018). Chronic nicotinamide riboside supplementation is well-tolerated and elevates NAD+ in healthy middle-aged and older adults. Nat. Commun..

[39] Cohen M.S. (2020). Interplay between compartmentalized NAD(+) synthesis and consumption: A focus on the PARP family. Genes. Dev..

[40] Weidele K., Kunzmann A., Schmitz M., Beneke S., Burkle A. (2010). Ex vivo supplementation with nicotinic acid enhances cellular poly(ADP-ribosyl)ation and improves cell viability in human peripheral blood mononuclear cells. Biochem. Pharmacol..

[41] Boyonoski A.C., Spronck J.C., Jacobs R.M., Shah G.M., Poirier G.G., Kirkland J.B. (2002). Pharmacological intakes of niacin increase bone marrow poly(ADP-ribose) and the latency of ethylnitrosourea-induced carcinogenesis in rats. J. Nutr..

[42] Hinshaw D.B., Lodhi I.J., Hurley L.L., Atkins K.B., Dabrowska M.I. (1999). Activation of poly [ADP-Ribose] polymerase in endothelial cells and keratinocytes: Role in an in vitro model of sulfur mustard-mediated vesication. Toxicol. Appl. Pharmacol..

[43] Martens M.E., Smith W.J. (2008). The role of NAD+ depletion in the mechanism of sulfur mustard-induced metabolic injury. Cutan. Ocul. Toxicol..

[44] Mol M.A., van de Ruit A.M., Kluivers A.W. (1989). NAD+ levels and glucose uptake of cultured human epidermal cells exposed to sulfur mustard. Toxicol. Appl. Pharmacol..

[45] Thompson B.C., Halliday G.M., Damian D.L. (2015). Nicotinamide enhances repair of arsenic and ultraviolet radiation-induced DNA damage in HaCaT keratinocytes and ex vivo human skin. PLoS ONE.

[46] Zhen A.X., Piao M.J., Kang K.A., Fernando P., Kang H.K., Koh Y.S., Yi J.M., Hyun J.W. (2019). Niacinamide Protects Skin Cells from Oxidative Stress Induced by Particulate Matter. Biomol. Ther..

[47] Grozio A., Mills K.F., Yoshino J., Bruzzone S., Sociali G., Tokizane K., Lei H.C., Cunningham R., Sasaki Y., Migaud M.E. (2019). Slc12a8 is a nicotinamide mononucleotide transporter. Nat. Metab..

[48] Schmidt M.S., Brenner C. (2019). Absence of evidence that Slc12a8 encodes a nicotinamide mononucleotide transporter. Nat. Metab..

[49] Ratajczak J., Joffraud M., Trammell S.A.J., Ras R., Canela N., Boutant M., Kulkarni S.S., Rodrigues M., Redpath P., Migaud M.E. (2016). NRK1 controls nicotinamide mononucleotide and nicotinamide riboside metabolism in mammalian cells. Nat. Commun..

[50] Palmer R.D., Elnashar M.M., Vaccarezza M. (2021). Precursor comparisons for the upregulation of nicotinamide adenine dinucleotide. Novel approaches for better aging. Aging Med..

[51] Dellinger R.W., Santos S.R., Morris M., Evans M., Alminana D., Guarente L., Marcotulli E. (2017). Repeat dose NRPT (nicotinamide riboside and pterostilbene) increases NAD(+) levels in humans safely and sustainably: A randomized, double-blind, placebo-controlled study. NPJ Aging Mech. Dis..

[52] Poljšak B., Kovač V., Milisav I. (2022). Current Uncertainties and Future Challenges Regarding NAD+ Boosting Strategies. Antioxidants.

[53] Kourtzidis I.A., Dolopikou C.F., Tsiftsis A.N., Margaritelis N.V., Theodorou A.A., Zervos I.A., Tsantarliotou M.P., Veskoukis A.S., Vrabas I.S., Paschalis V. (2018). Nicotinamide riboside supplementation dysregulates redox and energy metabolism in rats: Implications for exercise performance. Exp. Physiol..

[54] Nakahata Y., Sahar S., Astarita G., Kaluzova M., Sassone-Corsi P. (2009). Circadian control of the NAD+ salvage pathway by CLOCK-SIRT1. Science.

[55] Lin P., Bernstein I.A., Vaughan F.L. (1994). Failure to observe a relationship between bis-(beta-chloroethyl)sulfide-induced NAD depletion and cytotoxicity in the rat keratinocyte culture. J. Toxicol. Environ. Health.

[56] Mol M.E., de Vries R., Kluivers A.W. (1991). Effects of nicotinamide on biochemical changes and microblistering induced by sulfur mustard in human skin organ cultures. Toxicol. Appl. Pharmacol..

[57] Smith W.J., Gross C.L., Chan P., Meier H.L. (1990). The use of human epidermal keratinocytes in culture as a model for studying the biochemical mechanisms of sulfur mustard toxicity. Cell Biol. Toxicol..

[58] Sayer N.M., Whiting R., Green A.C., Anderson K., Jenner J., Lindsay C.D. (2010). Direct binding of sulfur mustard and chloroethyl ethyl sulphide to human cell membrane-associated proteins; implications for sulfur mustard pathology. J. Chromatogr. B Anal. Technol. Biomed. Life Sci..

[59] Sabnam S., Pal A. (2019). Relevance of Erk1/2-PI3K/Akt signaling pathway in CEES-induced oxidative stress regulates inflammation and apoptosis in keratinocytes. Cell Biol. Toxicol..

[60] Sabnam S., Rizwan H., Pal S., Pal A. (2020). CEES-induced ROS accumulation regulates mitochondrial complications and inflammatory response in keratinocytes. Chem. Biol. Interact..

[61] Tewari-Singh N., Gu M., Agarwal C., White C.W., Agarwal R. (2010). Biological and molecular mechanisms of sulfur mustard analogue-induced toxicity in JB6 and HaCaT cells: Possible role of ataxia telangiectasia-mutated/ataxia telangiectasia-Rad3-related cell cycle checkpoint pathway. Chem. Res. Toxicol..

[62] Brakedal B., Dolle C., Riemer F., Ma Y., Nido G.S., Skeie G.O., Craven A.R., Schwarzlmuller T., Brekke N., Diab J. (2022). The NADPARK study: A randomized phase I trial of nicotinamide riboside supplementation in Parkinson’s disease. Cell Metab..

[63] Sun X., Cao B., Naval-Sanchez M., Pham T., Sun Y.B.Y., Williams B., Heazlewood S.Y., Deshpande N., Li J., Kraus F. (2021). Nicotinamide riboside attenuates age-associated metabolic and functional changes in hematopoietic stem cells. Nat. Commun..

[64] Reiten O.K., Wilvang M.A., Mitchell S.J., Hu Z., Fang E.F. (2021). Preclinical and clinical evidence of NAD(+) precursors in health, disease, and ageing. Mech. Ageing Dev..

