# Supplementary material for: NAD+ Acts as a Protective Factor in Cellular Stress Response to DNA Alkylating Agents
Source: Cells. 2023 Oct 2;12(19):2396. doi: 10.3390/cells12192396 (PMC10572126; doi:10.3390/cells12192396)
Supplement: Supplementary file 1 [file cells-12-02396-s001.zip › cells-2601575-supplementary.pdf]

Supplementary information for

NAD$^+$ acts as a protective factor in cellular stress response to DNA alkylating agents

Joanna Ruszkiewicz[1] *, Ylea Papatheodorou[1], Nathalie Jäck[1], Jasmin Melzig[1], Franziska Eble[1], Annika Pirker[1], Marius Thomann[1], Andreas Haberer[1], Simone Rothmiller[2], Alexander Bürkle[1] * and Aswin Mangerich[1,3] *

[1] Molecular Toxicology Group, Department of Biology, University of Konstanz, 78457, Konstanz, Germany

[2] Bundeswehr Institute of Pharmacology and Toxicology, 80937, Munich, Germany

[3] Nutritional Toxicology, Institute Nutritional Science, University of Potsdam, 14469 Potsdam, Germany

***Correspondence:** joanna.ruszkiewicz@uni-konstanz.de, alexander.buerkle@uni-konstanz.de, mangerich@uni-potsdam.de

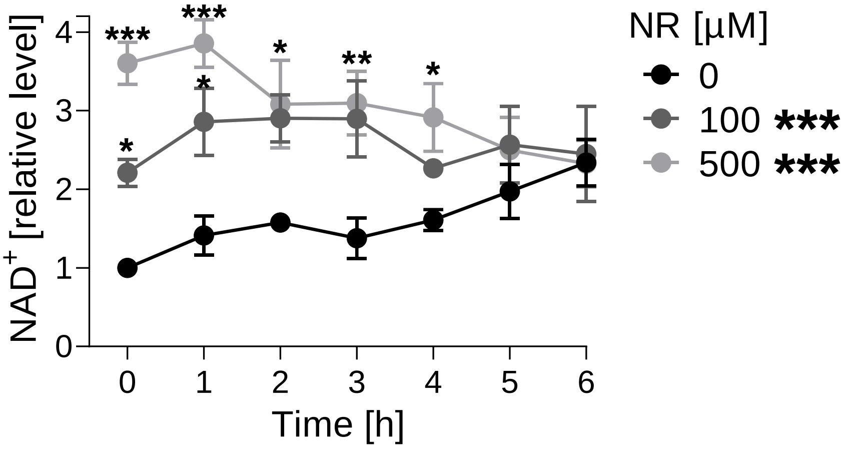

Figure S1: NR-dependent cellular NAD+ increase. HaCaT cells were exposed to NR for 3 h, next the growth medium was exchanged for fresh medium without NR, and cells were harvested at indicated time points. Cellular NAD+ levels were measured via enzymatic cycling assay and data were normalized to the control "0 µM" at 0 h. The results were expressed as mean ±SEM and analyzed by two-way ANOVA with Tukey's multiple comparisons test (n=4). *P<0.05, ** P<0.01, *** P<0.001.

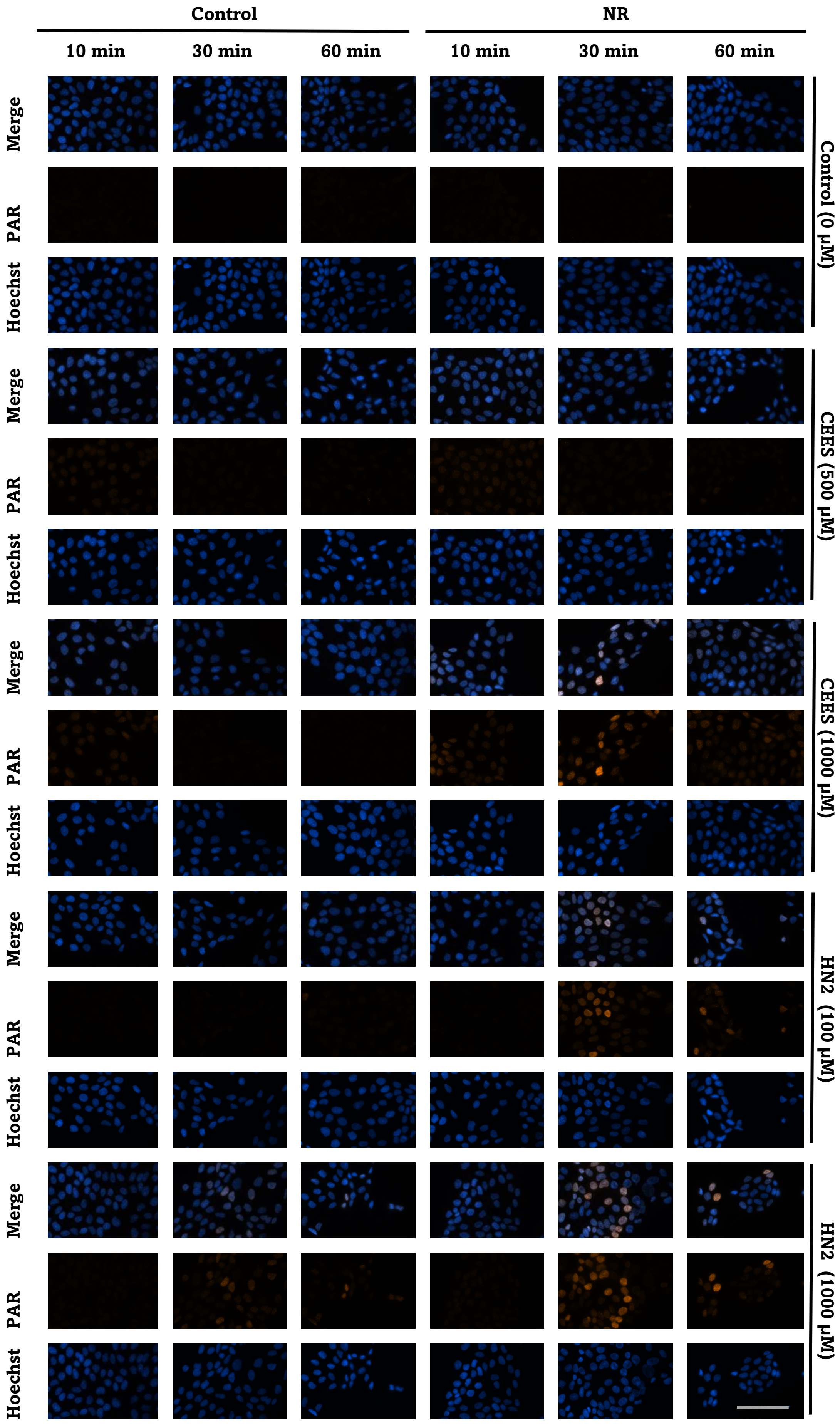

Control
NR
10 min
30 min
60 min
10 min
30 min
60 min
Merge
PAR
Hoechst
Control (0 μM)
CEES (500 μM)
CEES (1000 μM)
HN2 (100 μM)
HN2 (1000 μM)

Figure S2: NR elevates PAR levels during genotoxic stress. HaCaT cells were supplemented with 100 µM NR for 3 h and further treated with CEES or HN2 for 10, 30, or 60 min in PBS; "0 mM" refers to solvent control. At the end of each time point cells were fixed with ice-cold methanol, stained with anti-PAR antibody (10H) and DNA dye (Hoechst) and the signal was captured using an epifluorescence microscope. Scale bar indicates 100 µm.

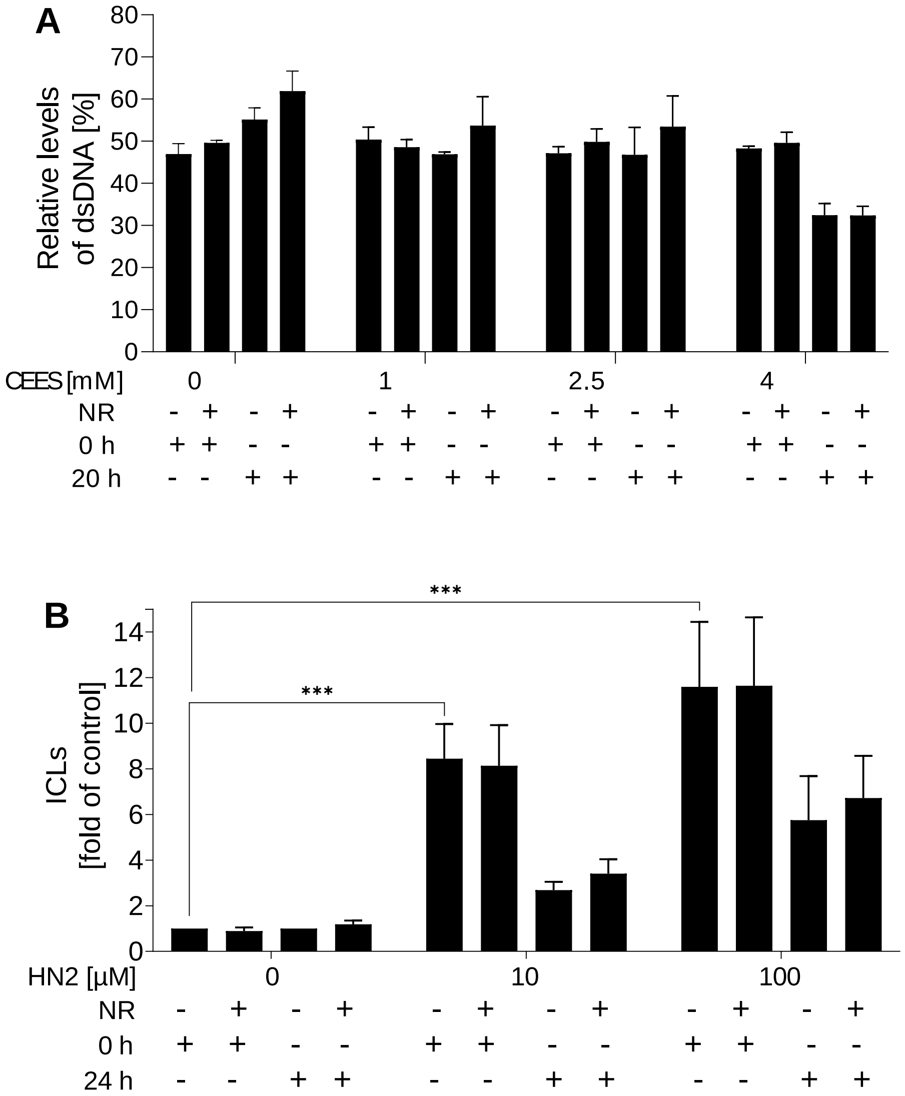

Figure S3: NR does not affect the formation and repair of DNA damage induced by alkylating agents. HaCaT cells were supplemented with 100 µM NR for 3 h and further treated with CEES (**A**, n=3), or HN2 (B, n=4) for 30 min in PBS. "0" refers to solvent control. Next cells were washed and incubated with fresh growth medium ±100 µM NR for 20 h (A) or 24 h (**B**), or harvested immediately (0 h). Induction of DNA strand breaks, which is inversely proportional to the relative levels of double-stranded DNA (dsDNA) remaining after alkaline unwinding, by CEES was measured via fluorimetric detection of alkaline DNA unwinding (FADU) assay (A), whereas induction of inter-strand crosslinks (ICLs) by HN2 was measured with reversed automated fluorimetric detection of alkaline DNA unwinding (rFADU) assay. Results were expressed as mean +SEM and analyzed by two-way ANOVA with Tukey's multiple comparisons test. *** P<0.001.

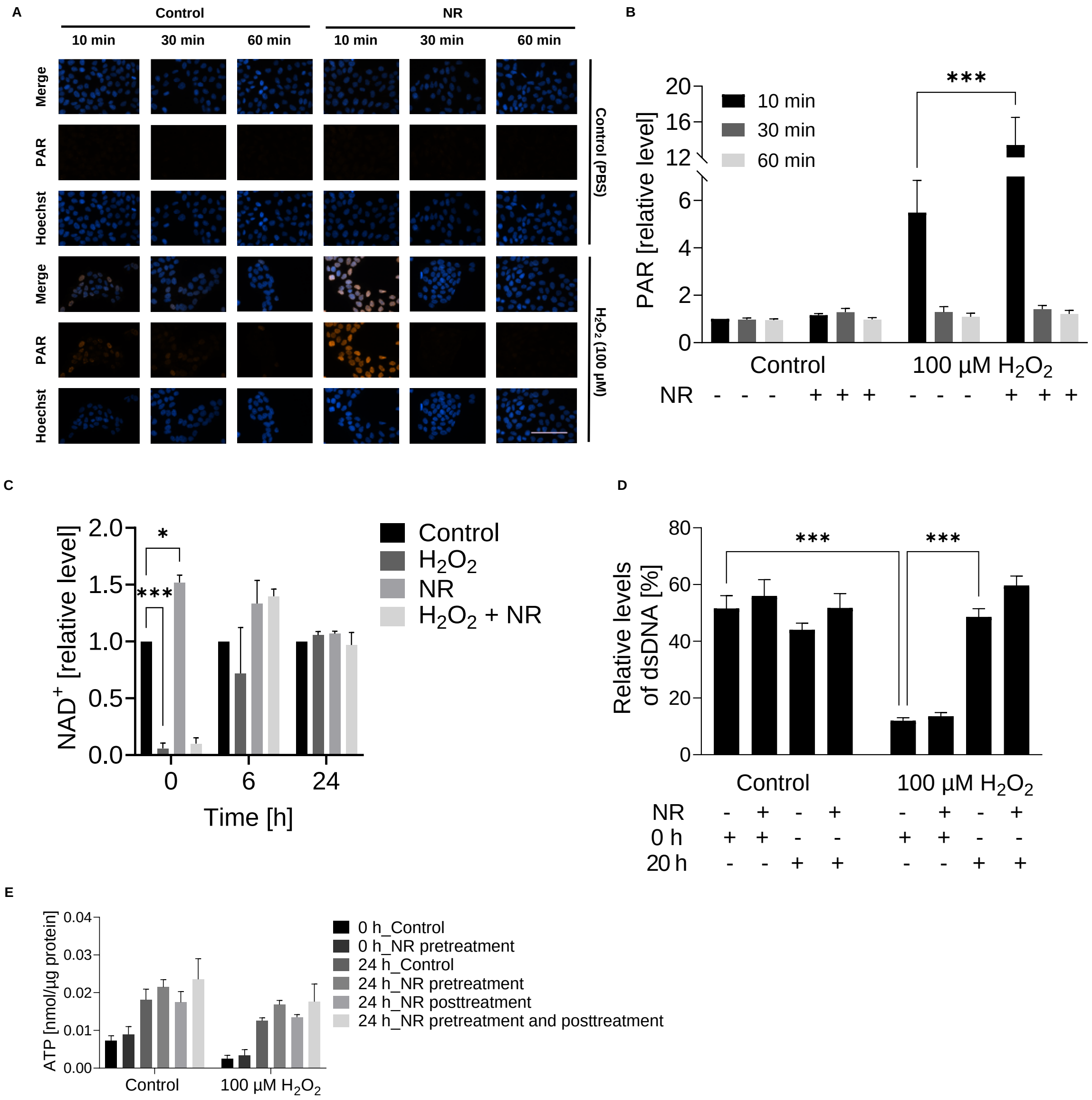

Figure S4: Effects of NR on H_2O_2 response in HaCaT. A: HaCaT cells were supplemented with 100 µM NR for 3 h and further treated with 100 µM H_2O_2 for 10, 30, or 60 min in PBS ("Control"). At the end of each treatment cells were fixed with ice-cold methanol, stained with anti-PAR antibody (10H) and DNA dye (Hoechst), and the signal was captured using an epifluorescence microscope. Scale bars indicate 100 µm. **B:** Images were automatically analyzed using KNIME software, and results were normalized to "Control" (10 min) (n=3). **C:** HaCaT cells were supplemented with 100 µM NR for 3 h and further treated with 100 µM H_2O_2 for 10 min. Next, cells were incubated in fresh growth medium with or without NR (up to 24 h) or harvested immediately (0 h). The NAD^+ levels were measured via enzymatic cycling assay, normalized to the total protein level measured by BCA, and expressed as fold change of "Control" at 0 h (n=2-3). **D:** Formation of DNA strand breaks was measured via fluorimetric detection of alkaline DNA unwinding (FADU) assay (n=3). **E:** ATP levels were measured via Cellular ATP Kit HTS and normalized to the total protein measured by BCA (n=3-4). Results were expressed as mean +SEM and analyzed by two-way ANOVA with Tukey's multiple comparisons test. *$P<0.05$, *** $P<0.001$.

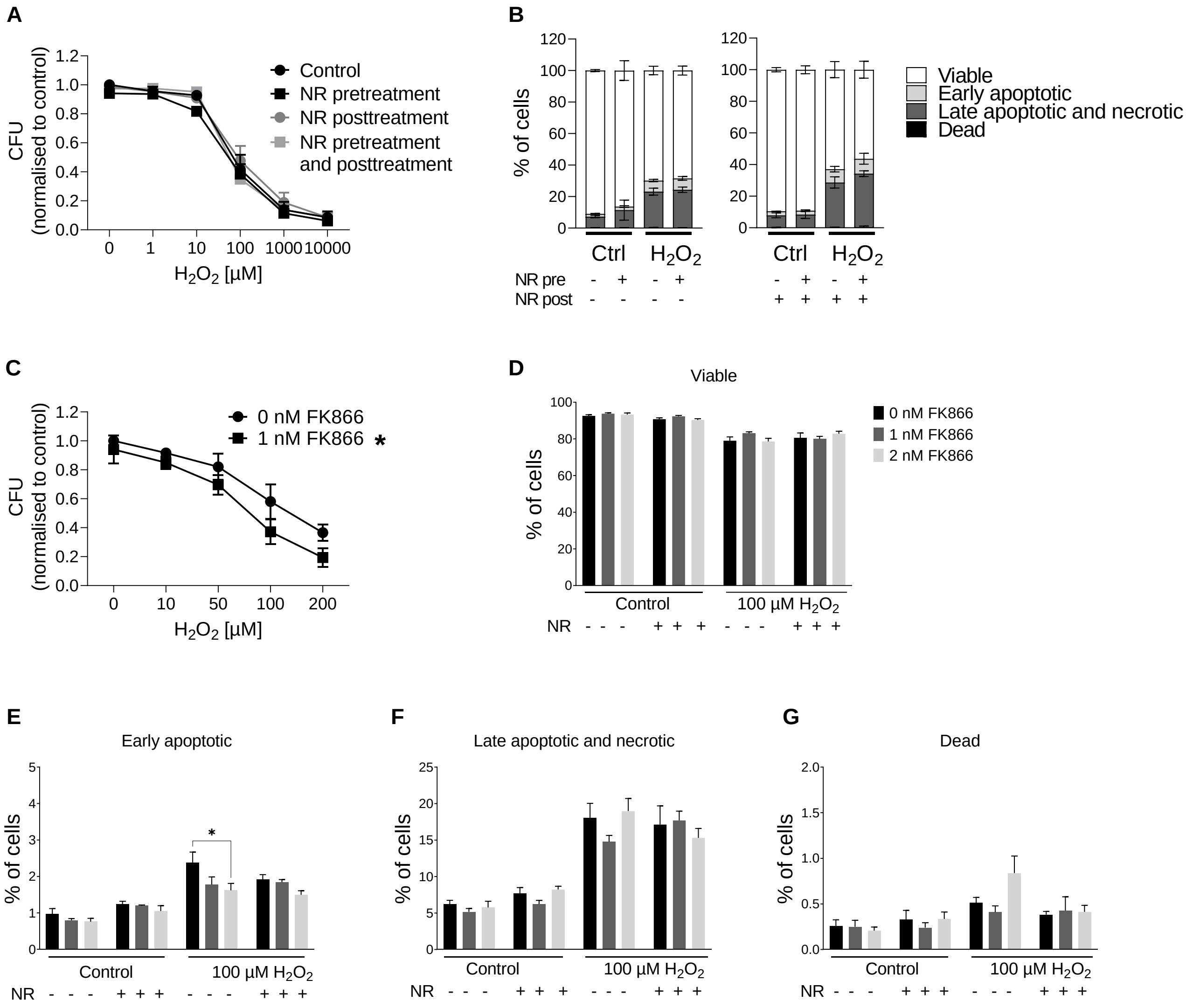

Figure S5: Effects of NR on H_2O_2 (geno)toxicity. HaCaT cells were supplemented with 100 µM NR for 3 h and further treated with H_2O_2 for 10 min. **A**: After treatment, cells were reseeded 1,000 cells per well in technical triplicates and incubated in fresh growth medium ±NR for 7 days. Then colonies were stained and counted. For each experiment, an average from technical replicates of the colony-forming unit (CFU) was calculated and normalized to "Control" (0 µM H_2O_2) (n=3). **B:** After treatment cells were incubated in fresh growth medium with or without NR for 24 h and cell death was analyzed via flow cytometry as described in Materials and methods (n=6), **C:** Cells were treated and analyzed like in (A), additionally FK866 was added to the culture, as described in Materials and methods (n=3); **D-G**: Cells were treated and analyzed via FACS like in (B), additionally FK866 was added to the culture, as described in Materials and methods (n=3-4). Results were expressed as mean ±SEM and analyzed by two-way ANOVA with Tukey's multiple comparisons test. *P<0.05.

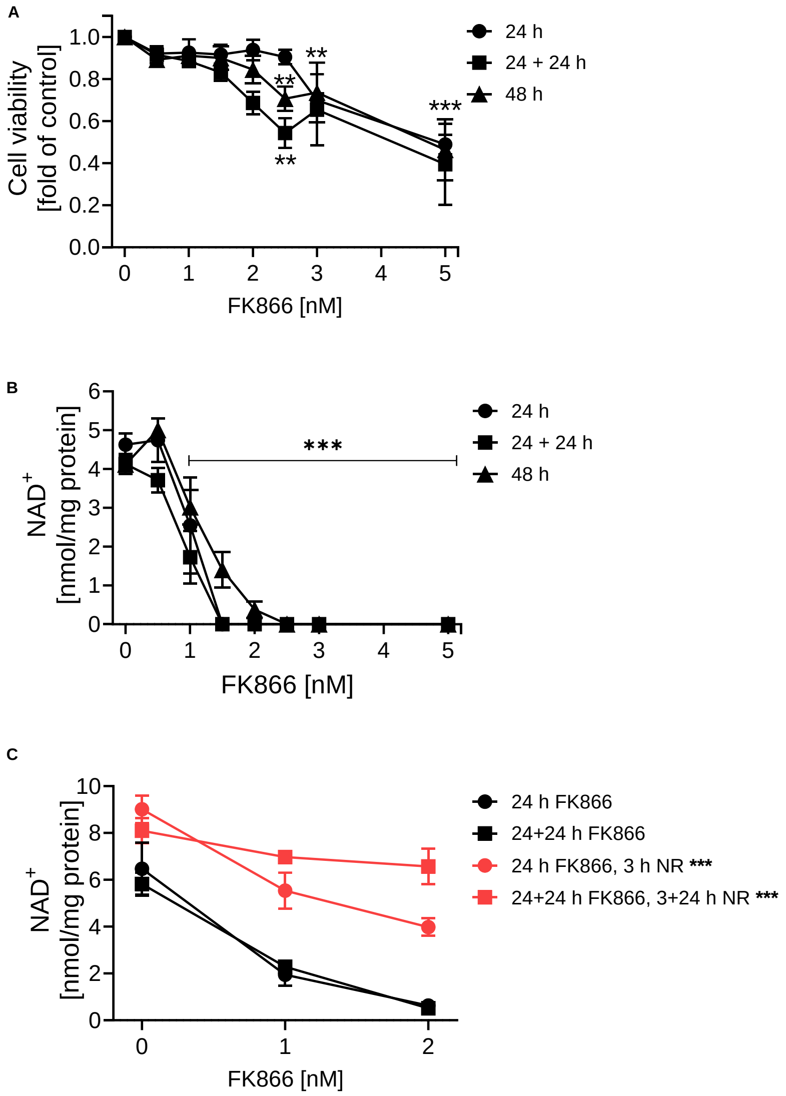

Figure S6: The effects of FK866 on HaCaT cells. HaCaT cells were treated with FK866 in 0.5% DMSO (0 nM FK866) in growth medium for 24 h or 48 h, additionally, the repeated exposure treatment (24+24 h) was applied, where fresh growth medium with FK866 was replaced after 24 h and cells were incubated for additional 24 h. **A:** Cell viability was measured via alamarBlue assay and results were normalized to “0 nM” (n=3). **B:** Cellular NAD^+ levels were measured via enzymatic cycling assay and normalized to the total protein level measured by BCA (n=3). **C:** HaCaT cells were treated with FK866 for 24 h and 24 + 24 h as in (A), additionally 100 μM NR was applied either 3 h before cell harvesting during 24 h FK866 exposure, or with the new growth medium (3+24 h) for 24 + 24 h FK866 exposure. Cellular NAD^+ levels were measured via enzymatic cycling assay and normalized to the total protein level measured by BCA (n=3). Results were expressed as mean ±SEM and analyzed by two-way ANOVA with Tukey’s multiple comparisons test. ** $P<0.01$, *** $P<0.001$.

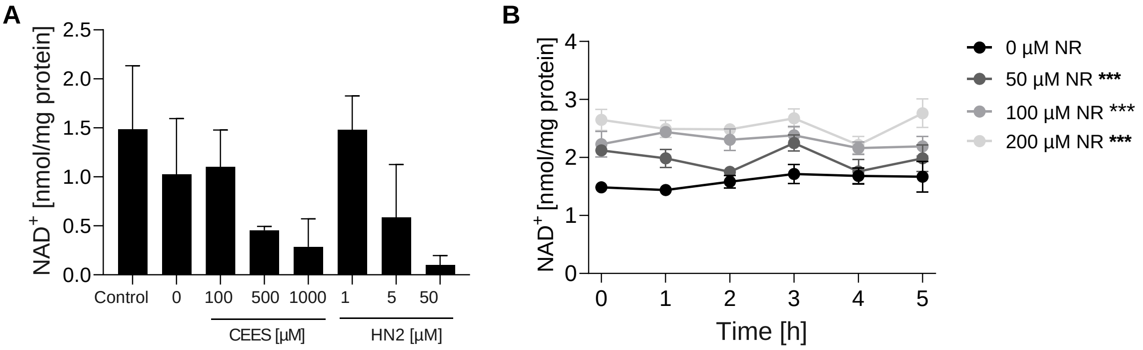

Figure S7: NAD⁺ levels upon mustards and NR treatments in THP-1 cells. A: THP-1 cells were exposed to CEES or HN2 in growth medium (“Control”) for 30 min; “0” refers to solvent control. Next, cells were washed and incubated with fresh growth medium for 24 h, when cells were harvested for NAD⁺ extraction (n=2). **B:** THP-1 cells were exposed to NR for 4 h, then the growth medium was replaced with fresh medium without NR, and cells were harvested up to 5 h later and cellular NAD⁺ was extracted (n=4). The NAD⁺ levels were measured via enzymatic cycling assay and normalized to the total protein level measured via BCA. The results were expressed as mean ±SEM and analyzed by two-way ANOVA with Tukey's multiple comparisons test. *** P<0.001.
